# Serum amyloid A is a positive acute phase protein in Russian sturgeon challenged with *Aeromonas hydrophila*

**DOI:** 10.1038/s41598-020-79065-9

**Published:** 2020-12-17

**Authors:** Mauricio Castellano, Valeria Silva-Álvarez, Marcio Aversa-Marnai, María Lamas-Bervejillo, Ignacio Quartiani, Alejandro Perretta, Andrea Villarino, Ana María Ferreira

**Affiliations:** 1 Unidad de Inmunología Asociada al Instituto de Química Biológica, Facultad de Ciencias - Área de Inmunología, Departamento de Biociencias, Facultad de Química, Universidad de la República, CP 11600 Montevideo, Uruguay; 2 Instituto de Investigaciones Pesqueras, Facultad de Veterinaria, Universidad de la República, CP 11300 Montevideo, Uruguay; 3 Sección Bioquímica y Biología Molecular, Facultad de Ciencias, Universidad de la República, CP 11400 Montevideo, Uruguay

**Keywords:** Animal biotechnology, Applied immunology, Biotechnology, Immunology, Applied immunology, Innate immunity

## Abstract

The immune system of sturgeons, one of the most ancient and economically valuable fish worldwide, is poorly understood. The lack of molecular tools and data about infection biomarkers hinders the possibility to monitor sturgeon health during farming and detect infection outbreaks. To tackle this issue, we mined publicly available transcriptomic datasets and identified putative positive acute-phase proteins (APPs) of Russian sturgeons that could be induced by a bacterial infection and monitored using non-invasive methods. Teleost literature compelled us to focus on five promising candidates: hepcidin, a warm acclimation associated hemopexin, intelectin, serum amyloid A protein (SAA) and serotransferrin. Among them, SAA was the most upregulated protein at the mRNA level in the liver of sturgeons challenged with heat-inactivated or live *Aeromonas hydrophila*. To assess whether this upregulation yielded increasing SAA levels in circulation, we developed an in-house ELISA to quantify SAA levels in sturgeon serum. Circulating SAA rose upon bacterial challenge and positively correlated with hepatic *saa* expression. This is the first time serum SAA has been quantified in an Actinopterygii fish. Since APPs vary across different fish species, our work sheds light on sturgeon acute-phase response, revealing that SAA is a positive APP with potential value as infection biomarker.

## Introduction

Sturgeons (Chondrostei: Acipenseridae) are one of oldest non-teleost Actinopterygii^1,2^. These fish are cultured for their meat and caviar and have therefore become a very valuable resource^3^. This has given rise to a growing aquaculture industry and with it, a renewed interest to understand sturgeon’s biology across multiple disciplines like evolutionary biology^4,5^, genomics^6,7^ and immunology^8–12^. However, sturgeons remain poorly studied, making research both challenging and indispensable for the growth of this sector^13^.

*Acipenser gueldenstaedtii* (Russian sturgeon) is the second most important species reared for caviar production, with more than 16 countries involved^3^. Uruguay successfully introduced and developed Russian sturgeon aquaculture and it is currently one of the top ten caviar producers worldwide^3^. However, Uruguayan farms are challenged by an increase in fish mortality during summer. We demonstrated this problem is linked to the induction of a chronic stress response that may be elicited by several factors, including the high water temperature, which negatively affects sturgeon innate immune system^10^. This paves the way for opportunistic pathogens like *Aeromonas hydrophila*^14^*,* which has been recognized as one of the major pathogens in sturgeon farming^11,15^. Furthermore, this problem leads to recurrent use of antibiotics, which may alter the normal fish microbiota promoting the selection of antibiotic-resistant strains^16^. To help tackle these challenges, in this study we search for infection serum biomarkers to monitor sturgeon´s health, which would contribute to prevent infection outbreaks and control the spread of diseases in farms.

Acute-phase proteins (APPs) are good candidates for this purpose since they are highly regulated at early stages of infections and can be measured in serum samples obtained by non-invasive methods^17–19^. Indeed, they comprise more than 200 biochemically and functionally diverse plasma proteins, predominantly synthesized in the liver and regulated by inflammatory cytokines (IL-6, IL-1β and TNF-⍺)^20^. APP serum levels rise or decline usually from 6 h to 3 days post-infection as the inflammatory cytokines are rapidly secreted by tissue-resident innate immune cells after sensing danger signals derived from injuries or infections^21^. After reaching the bloodstream, these proteins help fight pathogens, remodel damaged tissues and restore homeostasis. When danger signals disappear and the inflammatory reaction is resolved APPs return to their basal levels. However, if danger signals persist overtime, APP levels remain altered^22,23^.

According to their increase or decrease in serum concentration, APPs are classified as either positive or negative^24^. Positive APPs can be further classified as minor, moderate or major depending on whether their serum concentration rises twofold, between two and tenfold or more than tenfold, respectively^24^. Moreover, some major APPs can be induced over 100 to 1000-fold, making them great biomarkers of infection and/or inflammatory disorders for both human and veterinary animals^18,19,25^.

APPs are conserved among vertebrates, but proteins that behave as positive or negative APPs in mammals do not necessarily do so in fish, this is also valid across different fish species^26–30^. Regarding fish APPs, multiple orthologs have been identified in teleost and their gene expression and serum levels quantified using diverse infection models, including *A. hydrophila*^26,29,31–34^. Besides, studies have shown that teleost APPs have a different number of gene isoforms than their mammalian counterparts and that APPs are expressed not only in the liver but also in other immune relevant organs^29,35,36^. In the case of Russian sturgeon, a non-teleost fish, the major APPs induced by bacterial infections have not been identified yet. Furthermore, our knowledge on how sturgeon´s immune system deals with pathogens is poorly understood, in part, because molecular and genomic resources for this fish are scarce and species-specific reagents are almost non-existent. In addition, transcriptomic analyses were initially focused in gonads^37,38^, but more recently have been applied to immune relevant organs, like the spleen and head kidney, showing increased tissue expression of potential APPs (hepcidin, haptoglobin, transferrin and some complement components) after early infection with *A. hydrophila* or stimulation with Poly(I:C)^11,12,39–41^. Whether these proteins are rapidly upregulated in the liver, leading to higher serum levels remains unknown. Fortunately, the *Acipenser ruthenus* genome was recently published, and some APP sequences are now available in public DNA repositories^6,7^.

In this work, we aimed to identify positive APPs in Russian sturgeons that could serve as potential serum biomarkers to monitor fish health status in farms. To that end, we focused on five candidate proteins that are known to be upregulated upon infection or inflammatory challenge in fish species, including hepcidin (HEPC)^42,43^, the hemopexin named warm acclimation protein 65 kDa (HPX/WAP65-2)^44–47^, ntelectin (ITLN)^48–50^, serum amyloid A protein (SAA)^43,51–54^ and serum transferrin (TRFE)^55–57^. We successfully identified their coding sequences in Russian sturgeon and found that SAA was the most upregulated protein at the mRNA level in the liver of Russian sturgeons challenged with heat-inactivated or live *A. hydrophila*. Furthermore, we developed a sandwich ELISA to quantify SAA in sturgeon serum, which allowed us to demonstrate a positive correlation between liver *saa* mRNA and SAA serum levels. To our knowledge, this is the first study that has quantified serum SAA levels in an Actinopterygii fish, demonstrating that SAA plays a role as a positive APP and could be a valuable serum biomarker of infection in sturgeon.

## Results

### Identification of potential *A. gueldenstaedtii* APPs

**Figure 1:**
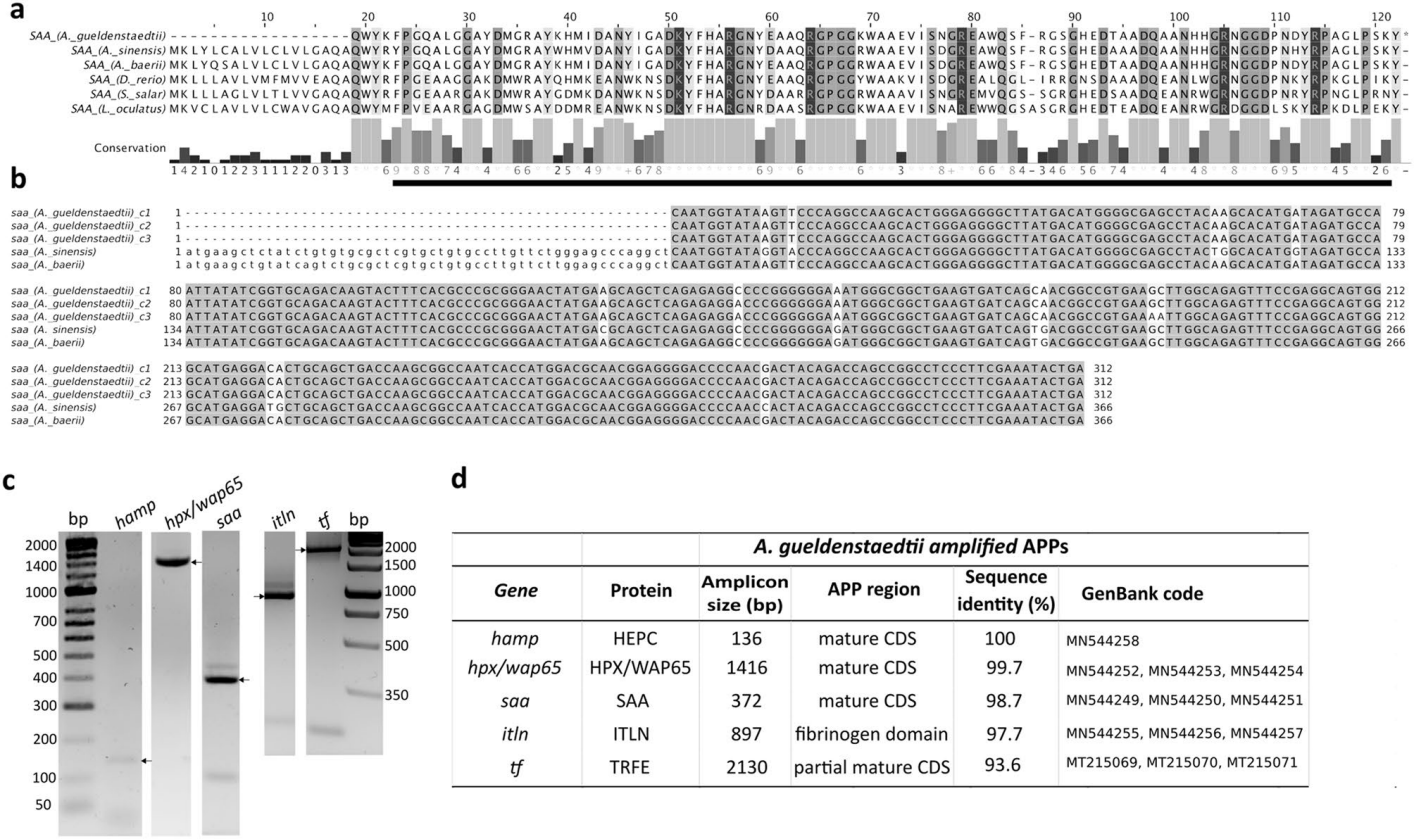
Teleost and sturgeon SAA alignment. (**a**) Amino acid sequence alignment of mature SAA from *A. gueldenstaedtii* (Russian sturgeon*,* consensus sequence), *A. sinensis* (Chinese sturgeon*,* translated using GenBank ID GETX01015093.1), *A. baerii* (Siberian sturgeon*,* translated from the transcript comp122687_c0_seq1^58^), *D. rerio* (Zebrafish*,* UniProtKB Q642J9), *S. salar*, (Atlantic salmon, UniProtKB B9EPA2) and *L. oculatus* (Spotted Gar*,* UniProtKB W5MIJ6) using Clustal Omega. Sequence identity is shown in gray scale and the specific SAA domain (Pfam 00,277) is underlined in black. (**b**) Nucleotide alignment of *saa* from *A. gueldenstaedtii* (clones c1-c3, GenBank IDs: MN544249, MN544250 and MN544251, without the leader sequence), with the entire *saa* coding sequences from *A. sinensis* (GenBank ID GETX01015093.1) and *A. baerii* (comp122687_c0_seq1^58^), using Clustal Omega. The leader sequence is shown in lower case letters and the sequence identity is represented in shades of grey. (**c**) Analysis by agarose gel electrophoresis of RT-PCR products corresponding to *A. gueldenstaedtii hamp, hpx/wap65*, *saa*, *itln* and *tf* amplicons, showing the expected amplicon size as indicated by black arrows. RT-PCR products were run on agarose gels (1.5%) in parallel with the Mw marker. For a better illustration, gel images were cropped from full gels and aligned using the Mw marker as a reference. The original gels are shown in Supplementary Figure S12. (**d**) *A. gueldenstaedtii* amplified products: the table shows the expected amplicon size, the APP region, the percentage of identity of each APP consensus sequence against the corresponding *A. baerii* sequence, and the GenBank access code of the different clones.

At the start of this study there were no sturgeon APP sequences available in public DNA repositories and transcriptomic studies had not been done in *A. gueldenstaedtii* immune-relevant tissues. Using a bioinformatics approach, we successfully identify the sequence of *A. gueldenstaedtii* HEPC, HPX/WAP65-2, ITLN, SAA and TRFE. To that end, we mined publicly available transcriptomic datasets (unigenes) of *Acipenser baerii*^58^ and *Acipenser sinensis*^9^ using the Blastp algorithm and *Danio rerio* APPs as the query. In both sturgeon species, we identified HEPC, ITLN, SAA and TRFE homologs (Supplementary Table S1). Their identity was further verified by performing Blastp against the NCBInr database and by identifying conserved protein domains (CPD) using InterPro^59^ or SMART^60^. *Acipenser baerii* HPX/WAP65-2 sequence was kindly provided by Dr. Denise Vizziano (unigene GICD01044135.1^61^). The alignment of *A. baerii*, *A. sinensis, Lepisosteus oculatus*, *D. rerio* and *Salmo salar* APP sequences confirmed that these proteins are highly conserved among distantly related Actinopterygii fish as shown for SAA (Fig. 1a), HEPC, ITLN, TRFE as well as for HPX/WAP65 (Supplementary Figure S1a–S4a, respectively). Moreover, we found that *A. baerii* and *A. sinensis* APPs have almost identical nucleotide coding sequences despite having diverged more than 120 million years ago^2^ (Fig. 1b, Supplementary Figure S1b–S4b). Thus, given that *A. baerii* and *A. gueldenstaedtii* are closely related^2,5^, it was possible to design specific primers to amplify by RT-PCR the coding sequences of *A*. *gueldenstaedtii* HEPC, HPX/WAP65-2, ITLN, SAA and TRFE. For all *A. gueldenstaedtii* APPs studied, amplification products matched the expected amplicon size (Fig. 1c) and, as expected, their nucleotide sequences were highly similar to those of *A. baerii* and *A. sinensis* (Fig. 1b, Supplementary Figure S1b–S4b). For each APP, various clones were sequenced detecting single nucleotide variations for all APPs, except for HEPC (Fig. 1b,d, and Supplementary Figure S2b–S4b). These variations may be due to duplication events or different allelic variants considering that *A. gueldenstaedtii* is a tetraploid species^5,62^.

### SAA is transcriptionally induced in the liver of *A. gueldenstaedtii* following *A. hydrophila* challenge

**Figure 2:**
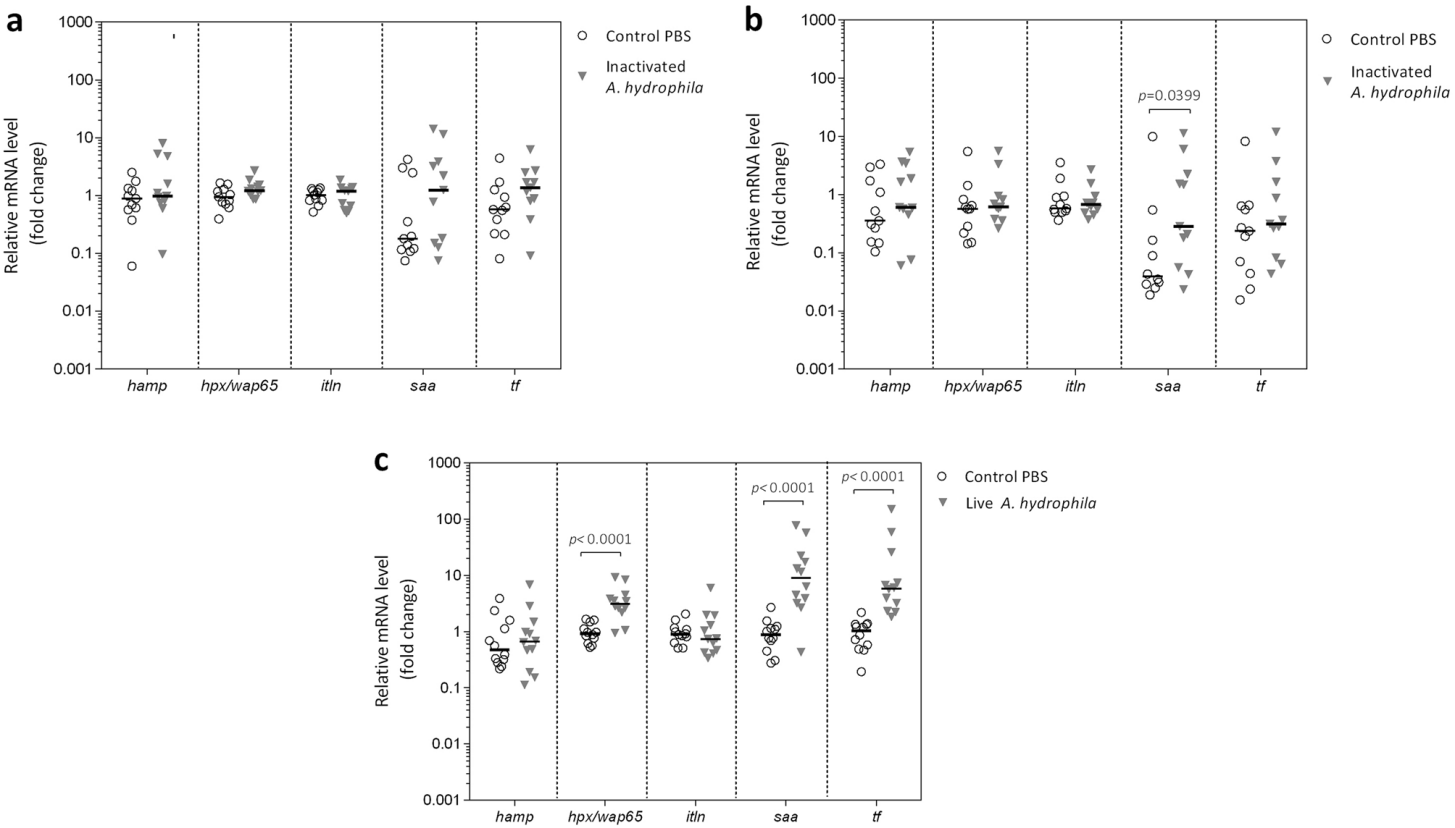
Liver APPs expression of *A. gueldenstaedtii* challenged with heat-inactivated or live *A. hydrophila.* Experiment with heat-inactivated bacteria: juvenile Russian sturgeons were ip challenged with heat-inactivated *A. hydrophila* or sterile PBS (control PBS), and liver APPs expression analysed at 1 dpc (**a**) or 3 dpc (**b**). Experiment with live bacteria: juvenile Russian sturgeons were ip challenged with live *A. hydrophila* or sterile PBS (control PBS), and liver APPs expression analysed 3 dpc (**c**). In both experiments the liver expression of APP genes was studied by RT-qPCR. Results are expressed as the increase (fold change) in the mRNA level, relative to the corresponding control group, determined with the 2^-ΔΔCT^ method using *gapdh* as housekeeping gene. Graphs show individual APP fold changes and their median value, illustrating the biological variability and the lack of normal distribution across samples. Data were transformed to meet normality (using the inverse square root or logarithmic transformation for the challenge experiments with heat-inactivated or live bacteria, respectively) and then the unpaired Student´s t-test with Welch's correction was applied to compare treated and control groups. Statistical significances (*p*-value) are shown.

To investigate which *A. gueldenstaedtii *APP candidates could constitute a promising infection biomarker, we evaluated by RT-qPCR their liver relative gene expression at 1 and 3 days post-challenge (dpc) with heat-inactivated *A. hydrophila* (Fig. 2). As shown in Fig. 2a, liver mRNA levels of all APPs studied remained unchanged across treatment groups at 1 dpc. However, at 3 dpc, *saa* mRNA levels exhibited an increment (sevenfold) in challenged sturgeons compared to mock-challenged controls, while the rest of APP candidates remained unaltered (Fig. 2b). These results suggested that in the assayed conditions *A. hydrophila* challenge elicited a mild acute-phase response in *A. gueldenstaedtii*, which becomes detectable after 3 dpc. In order to verify these results using a stronger inflammatory condition, we performed a similar analysis in sturgeons challenged with live *A. hydrophila* (Fig. 2c). In this model, fish behaviour, weight and mortality remained unchanged. However, at 3dpc, challenged fish showed increased *hpx*/*wap65-2* (threefold)*, saa* (tenfold) and *tf* (sixfold) mRNA levels in the liver in comparison with control sturgeons. Besides, histological analysis of liver samples showed that heat-inactivated and live *A. hydrophila* induced leukocyte infiltration in the portal space and hepatocyte alterations (vesiculation and pyknotic nuclei), which are compatible with the development of an acute inflammatory response^63^ (Figure S5). Altogether, results suggested that SAA was the most promising positive APP among the assayed candidates in *A*. *gueldenstaedtii*. Furthermore, tissue expression profile of unchallenged fish showed that the liver is the main expression site of *A*. *gueldenstaedtii saa,* with transcript levels up to 1500 times higher than those in the spleen, head kidney, gills and brain (Figure S6).

### Putative B-cell epitopes identified in *A*. *gueldenstaedtii* SAA are highly similar to those in human SAA

**Figure 3:**
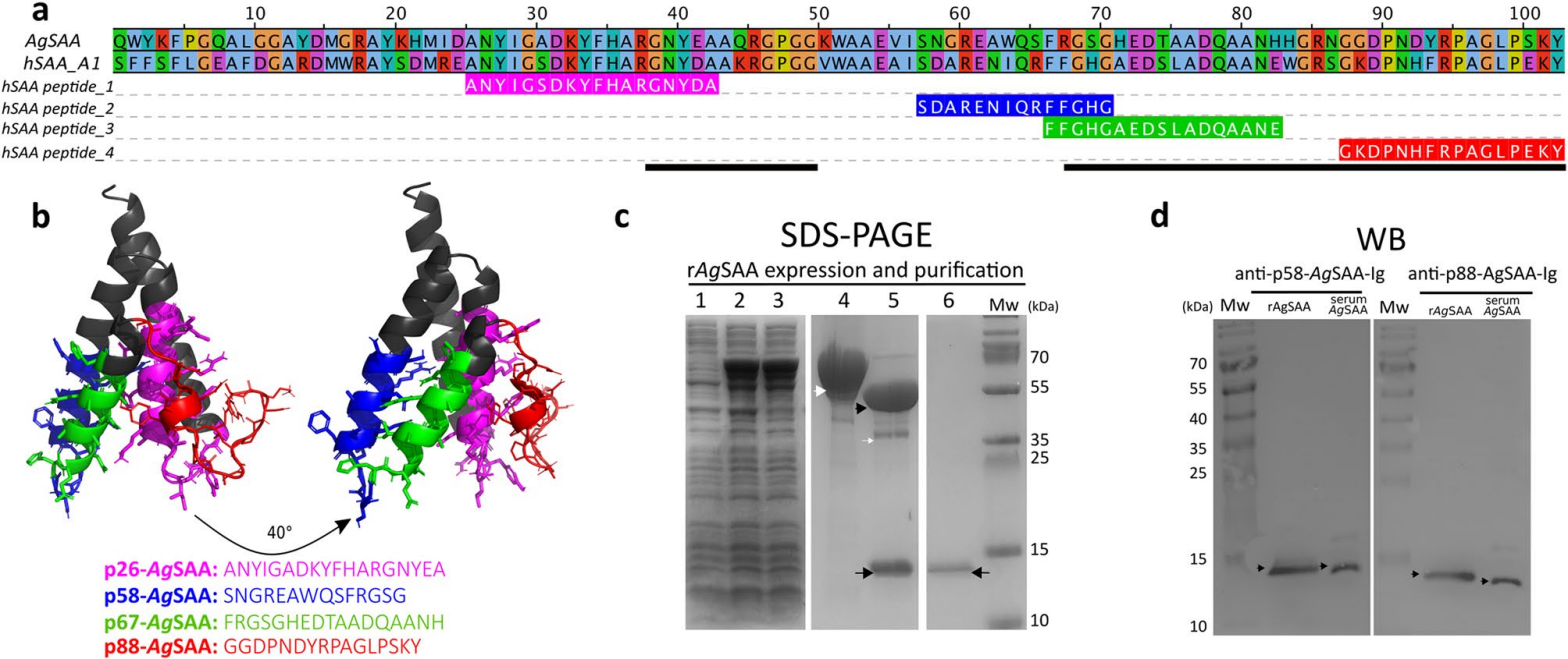
Identification of putative B-cell epitopes to the serum *Ag*SAA immunodetection. (**a**) Alignment of mature *Ag*SAA and hSAA1 (UniProtKB: P0DJI8) with four hSAA1 derived peptides (peptides 1–4)^64^. *A. gueldenstaedtii* homologues to these peptides were named p26-*Ag*SAA (aa 26–43), p58-*Ag*SAA (aa 58–71), p67-*Ag*SAA (aa 67–83) and p88-*Ag*SAA (aa 88–103). Black bars show putative *Ag*SAA B-cell epitopes predicted in silico (Bepipred algorithm). (**b**) *Ag*SAA structure model built with mSAA (PDB: 4Q5G) as template (SWISS-MODEL). Peptides p26-*Ag*SAA (pink), p58-*Ag*SAA (blue), p67-*Ag*SAA (green) and p88-*Ag*SAA (red) are indicated. Note that p58-*Ag*SAA and p88-*Ag*SAA are exposed at opposite sides of the protein. (**c**) Expression and purification of r*Ag*SAA in *E. coli.* Coomassie-stained SDS-PAGE-TRICINE corresponding the total fraction of an uninduced culture (lane 1), the total and soluble fractions of the IPTG-induced culture (lanes 2 and 3), the affinity-purified His-MBP-*Ag*SAA (lane 4), TEV-digested His-MBP-*Ag*SAA (lane 5) and pure r*Ag*SAA obtained after negative purification by IMAC followed by amylose affinity chromatography (lane 6). The band corresponding to His-MBP-*Ag*SAA (54.5 kDa, white head arrow), His-MBP (43.1 kDa, black head arrow), TEV_SH_ (white arrow) and the band corresponding to r*Ag*SAA (11.4 kDa, black arrow) are indicated. Samples were run on three separated gels. The regions of interest in each gel were cropped, aligned using the Mw marker as a reference. The original gels are shown in Supplementary Figure S13. (**d**) Detection of r*Ag*SAA and native *Ag*SAA by Western blot. r*Ag*SAA, sturgeon serum and prestained Mw marker were run on SDS-PAGE gels and transferred to PVDF membranes. For blotting, membranes were cut and incubated separately with anti-p58-*Ag*SAA-Ig (dilution of 1:4000, 150 s exposure time) or anti-p88-*Ag*SAA-Ig (dilution 1:1500, 150 s exposure time). Figure shows the merge image of chemiluminescent and the prestained Mw marker images. Black head arrows indicate the signal band corresponding to *Ag*SAA.

To demonstrate that *A*. *gueldenstaedtii* SAA (*Ag*SAA) behaves like an APP in Russian sturgeon, in addition to the observed increment in liver *saa* mRNA, circulating *Ag*SAA protein levels should raise in response to acute inflammation. Therefore, we set out to obtain antibodies against *Ag*SAA and design a sandwich ELISA to quantify native *Ag*SAA in serum samples. To identify putative *Ag*SAA B-cell epitopes, we aligned the *Ag*SAA sequence together with human SAA1 (hSAA1) and four hSAA1 peptides which are known to be immunogenic^64^ (Fig. 3a). Given that *Ag*SAA and *h*SAA1 sequences showed a 65% of identity, it was possible to identify the corresponding four *Ag*SAA peptides with homology to those present in hSAA1. These peptides, named p26-*Ag*SAA (spanning residues 26–43), p58-*Ag*SAA (spanning residues 58–71), p67-*Ag*SAA (spanning residues 67–83) and p88-*Ag*SAA (spanning residues 88–103), were 89%, 57%, 65% and 75% identical to hSAA1 peptides. Moreover, in silico B-cell epitope prediction algorithm determined two immunogenic regions within the *Ag*SAA sequence that partially or completely covered these peptides (Fig. 3a, see black bars). To assess the structure and surface exposure of these peptides, *Ag*SAA was modelled using mouse SAA3 (mSAA3) which had a 60% sequence identity with respect to *Ag*SAA (Supplementary Figure S7a) and was predicted as the best template using HHblits. Alignment of modelled *Ag*SAA with hSAA1 and mSAA3 showed that all three proteins have an almost identical 3D conformation containing the conserved 4-helix bundle structure shared by all SAA family proteins^65^ (Supplementary Figure S7b). This structural conservation implies that all four identified *Ag*SAA peptides could be promising B-cell epitopes. Since sandwich ELISA design requires that capture and detection antibodies bind to distantly located epitopes to prevent mutually exclusive interactions, we selected p58-*Ag*SAA and p88-*Ag*SAA to raise polyclonal antibodies in rabbits (Fig. 3b, Supplementary Figure S7c).

### A sensitive sandwich ELISA for *Ag*SAA was developed and used to quantify native *Ag*SAA in sturgeon serum

For this development, we first prepared recombinant *Ag*SAA (r*Ag*SAA) to purify by immunoaffinity chromatography the antigen-specific immunoglobulin (Ig) fraction of the rabbit antisera (anti-p58-*Ag*SAA-Ig and anti-p88-*Ag*SAA-Ig) and to build the ELISA standard curve. To that end, mature *Ag*SAA (without signal peptide) was overexpressed in *E. coli* as a chimeric protein with an N-terminal His-Maltose Binding Protein-tag (His-MBP-tag), which aided expression and purification steps (Fig. 3c). His-MBP-r*Ag*SAA was successfully produced as a soluble protein with an apparent Mw of 54.5 kDa. Then, the MBP-tag was removed after digestion with TEV_SH_ protease, which yield the expected soluble r*Ag*SAA and His-MBP-tag (theoretical Mw 43.1 kDa). After successive purifications steps, pure, tag-free and soluble r*Ag*SAA was successfully obtained, which behaves as an 8.7 kDa molecule in SEC, suggesting it is a monomer in the assayed conditions (Supplementary Figure S8). The r*Ag*SAA identity was confirmed by MALDI TOF/TOF MS and MASCOT searches using a custom database containing sturgeon translated transcriptomes (Supplementary Figure S9). Regarding antisera purification, both anti-p58-*Ag*SAA and anti-p88-*Ag*SAA antisera were immunoaffinity purified. The specificity of the obtained Ig fractions evaluated by WB showed that they were both able to recognize r*Ag*SAA and a unique sturgeon serum component compatible with native *Ag*SAA (Fig. 3d). Finally, using the prepared reagents, we developed a sandwich ELISA to detect *Ag*SAA in sturgeon serum, which employed anti p58-*Ag*SAA-Ig as capture antibody, biotinylated anti p88-*Ag*SAA-Ig as detection antibody and r*Ag*SAA as standard. This ELISA showed a good sensitivity detecting 2 ng/ml of r*Ag*SAA in binding buffer (Supplementary Figure S10a). However, 0.4 μg/ml of *Ag*SAA was the detection limit in serum due to the interference caused by serum components (matrix effect), which was overcome by a 1:200 serum dilution (Supplementary Figure S10b). This sensitivity was good enough to detect native *Ag*SAA in all assayed serum samples.

### *Ag*SAA is a positive APP and a valuable infection biomarker

**Figure 4:**
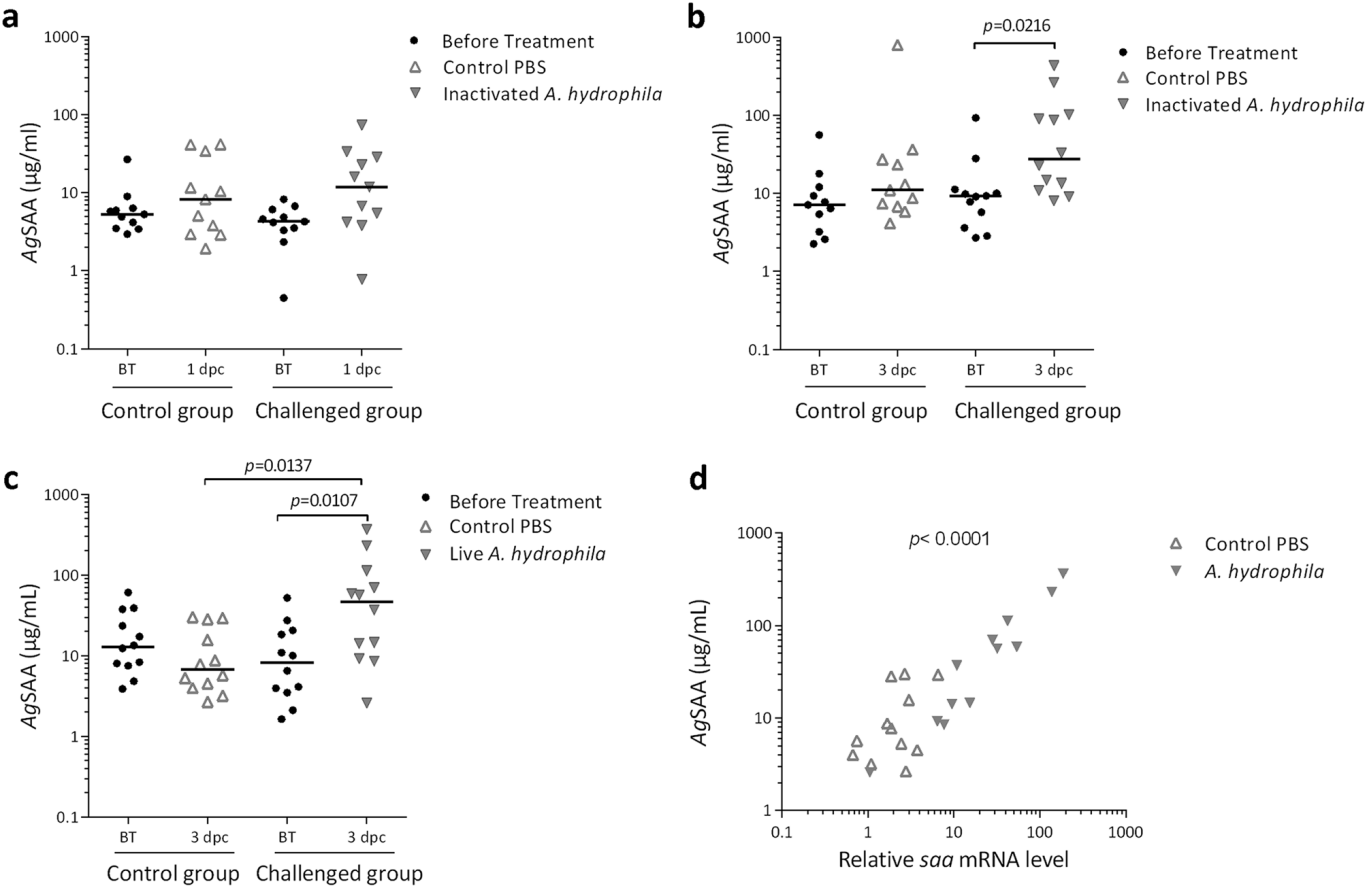
Serum *Ag*SAA levels in *A. gueldenstaedtii* challenged with heat-inactivated or live *A. hydrophila.* Experiment with heat-inactivated bacteria: juvenile Russian sturgeons were ip challenged with heat-inactivated *A. hydrophila* or sterile PBS (control PBS), and serum *Ag*SAA levels analysed at 1 dpc (**a**) or 3 dpc (**b**). Experiment with live bacteria: juvenile Russian sturgeons were ip challenged with live *A. hydrophila* or sterile PBS (control PBS), and serum *Ag*SAA levels analysed at 3 dpc (**c**). Individual and median values of serum *Ag*SAA levels (μg/ml) are indicated. Data were transformed to meet normality (using the inverse square root or logarithmic transformation for the challenge with heat-inactivated and live bacteria trial, respectively) and afterwards the one-way ANOVA with post-hoc Tukey´s test was applied for multiple comparisons. Statistical significances (*p*-value) are shown. The correlation between serum *Ag*SAA levels and relative liver *saa* mRNA levels (2^^-ΔCT^, determined using *gapdh* as housekeeping gene) is shown in (**d**). Statistical significance (*p*-value) analysed by Spearman test is indicated. This correlation was also found when *saa* mRNA levels were determined using *act-b* as housekeeping gene.

Using the optimized ELISA we quantified serum *Ag*SAA in sturgeons challenged with heat-inactivated or live *A. hydrophila* to establish if this protein behaves like a positive APP in our experimental models (Fig. 4). Challenge with heat-inactivated bacteria did not induce alterations in *Ag*SAA serum levels at 1 dpc (Fig. 4a). In contrast, at 3 dpc, serum *Ag*SAA levels increased threefold, on average, over their basal levels (Fig. 4b). This increment was not observed in the control group, suggesting that heat-inactivated bacteria induced a weak *Ag*SAA response. Furthermore, as expected, *Ag*SAA response was stronger in sturgeons challenged with live *A. hydrophila*. Indeed, at 3 dpc, serum *Ag*SAA protein increased sixfold over their basal levels and was up to 7.0-fold higher than values corresponding to the control group (Fig. 4c). Furthermore, serum *Ag*SAA concentrations positively correlated with liver *saa* mRNA levels in both, inactivated and live *A. hydrophila* challenged sturgeons, indicating that *Ag*SAA is mainly synthesized in the liver and could be a valuable infection biomarker (Fig. 4d and Supplementary Figure S11, respectively).

## Discussion

Farming conditions are usually stressful for fish, weakening their defences and threatening fish welfare. In a previous study, we demonstrated that long-term exposure to summer temperature causes chronic stress and alterations in innate immune components in farmed Russian sturgeons, favouring opportunistic infections^10^. In this context, sturgeon aquaculture and caviar production is challenging because fish sexual maturity takes several years, raising the need for novel tools to monitor fish health status and diminish the economic impact of infection outbreaks^66^. However, molecular biomarkers for sturgeon health screening are not available yet. This work analysed the potential of five proteins, described as APPs in some teleosts, and whose upregulation would strengthen natural defences making them good infection biomarkers^18,19^. These proteins were HEPC, HPX/WAP65-2 and TRFE, which regulate iron availability in the blood contributing to limit pathogen establishment and multiplication^47,56,57,67^, and ITLN and SAA, which behave as soluble receptors for bacteria and might mediate their agglutination and/or opsonization^28,68,69^. The coding sequence of these putative APPs was not available in public databases at the beginning of this investigation, although various *Acipenser* transcriptomes were published in the last years. By using bioinformatics tools, we found the coding sequence of HEPC, HPX/WAP65-2, TRFE, ITLN and SAA in *A. baeri* and *A. sinensis* public transcriptomes. Furthermore, because the remarkable sequence identity of these putative APPs among sturgeons, we could identify the corresponding sequences in *A. gueldenstaedtii*, which were also very similar to those found in *A. baeri* and *A. sinensis* (93.6–100%). Our findings extend recently published data of HEPC and TRFE in other *Acipenser* species while providing novel information on sturgeon HPX/WAP65-2, ITLN and SAA. The HEPC coding sequence that we found in *A. baeri* transcriptome agrees with that derived from the characterization of *A. baeri* HEPC gene (*hamp*)^70^, *A. dabryanus* mature HEPC^39^ and two *A. ruthenus* hepcidin-like proteins (GenBank XM_034056050 and XM_034056903.2). *Acipenser baeri hamp* showed a closer genetic relationship to tetrapodian orthologs than to teleostean orthologs, suggesting that chondrostean HEPC may be an evolutionarily ancestral form, which would have evolved into extant hepcidins present in tetrapods and teleosts^70^. Teleosts have two functionally different HEPC, represented by two or more isoforms, which are specialized in distinct functions, with *hamp1* being the major regulator of iron metabolism and *hamp2* having a preponderant antimicrobial role^42^. However, in *Acipenser* these two functions would be represented in a single gene, with longer total intron length than any of the described teleost hepcidin genes, being a structural feature that suggests a role in gene evolution and expression regulation^70^. Regarding TRFE, we identified three *tf* sequences in *A. gueldenstaedtii* showing more than 91% of identity with *A. ruthenus* TRFE 2, 3 and 8 isoforms, which are the ones completely sequenced among the eight isoforms (GenBanK codes XP_033894951.1, QHQ72345.1, QHQ72350.1^71^). Phylogenetic analysis of *A. ruthenus* transferrins showed that they formed a discrete well-supported cluster with serotransferrin-like proteins from other fish and that a non-accelerated evolution of transferrins would have occurred in fish compared to tetrapods^71^. In *A. bester*, a TRFE (serotransferrin 2-like isoform) has also been described as one of the three most expressed genes in the liver, but its coding sequence has not been deposited in GenBank^40^.

Among all selected APP candidates, HEPC and ITLN were not transcriptionally upregulated in the liver of heat-inactivated or live *A. hydrophila* challenged Russian sturgeons. The current data on *Acipenser* species reveal contrasting results for the regulation of *hamp* expression in the liver during infection with Gram-negative bacteria. While *hamp* was not upregulated after intraperitoneal bacterial challenge of *A. baerii*^70^ or *A. gueldenstaedtii* (this work), it exhibited an increase in *A. dabryanus* challenged by bath immersion, a route that mimics natural infection^39^. These dissimilar results may be due to differences in the route of infection and/or in the pathogenicity of the bacterial strains used. Therefore, further experiments are needed to determine whether *hamp* behaves like a positive APP in *Acipenser*. Regarding ITLNs, the scenario is more complex because they belong to a family of X-type lectins, comprising various isoforms in vertebrates, including fish. Moreover, even ITLN amino acid sequences in different species are conserved their expression patterns and functions differ considerably among and within species^69^. In teleosts, the major family member expressed in the liver might be species-specific and has been only documented in a few fish species, such as *D. rerio* and *Ictalurus punctatus*. In *D. rerio* at least five ITLN isoforms are expressed in adult tissues, with *itln*-3 being the isoform most expressed and upregulated in the liver upon infection with *A. salmonicida* and *Mycobacterium marinum*^72,73^*.* In *I. punctatus*, two major ITLN genes were identified, and *itln-2*, but not *itln-1*, was found to be highly expressed and upregulated in the liver after *E. ictaluri* infection^49^. We identified an *A. gueldenstaedtii itln* isoform that was more similar at the protein level to *D. rerio itln-2* (66.1–67.2% of identity) than to *D. rerio itln-3* or *I. punctatus itln* isoforms (between 50 and 61.5% of identity). This sturgeon *itln* was not upregulated in the liver upon bacterial infection suggesting that it does not behave as a positive APP. However, additional *A. gueldesntaedtii itln* isoforms in the liver might exist because the publicly transcriptome data available at the beginning of this work and used for *itln* identification derived from other tissues, and the designed primers might have not been able to hybridize with hepatic *itln* isoforms. However, taken advantage of recent genomic data, we found in *A. ruthenus* only two intelectin-1 like sequences that were highly similar to the one identified in *A. gueldesntaedtii* (between 96 and 99%) during this work^6^. This makes unlikely that the primers designed in this work were unable to quantify all sturgeon *itln* isoforms.

Three of the putative APPs selected in this study, HPX/WAP65-2, TRFE and SAA, were upregulated at mRNA level in the liver of sturgeons challenged with live *A. hydrophila*, supporting that they behave as positive APPs in *A. gueldenstaedtii*. HPX/WAP65-2 is one of the members of the HPX/WAP65 family in fish, whose mammalian orthologs are hemopexins with hemo scavenger activity. While both HPX/WAP65 isoforms might be involved in the fish response to temperature acclimation, *hpx/wap65-2* seems to work as an immune-related gene as well, being highly and positively induced during bacterial infection in various teleosts^44,74^. We identified an *A. gueldenstaedtii hpx/wap65-2* sequence that showed 99% similarity to a recently annotated *A. ruthenus* hemopexin-like protein (GenBank XM_034010562.2:49–1416). Since *hpx/wap65-2* upregulation was modest in our model (threefold), this result indicates that this hemopexin would act as a moderate APP in this chondrostean fish. In comparison with HPX/WAP65-2, TRFE seems to be a more sensitive APP in sturgeon because hepatic *tf* expression was sixfold increased upon bacterial challenge. Furthermore, this early *tf* upregulation observed at 3 dpc in *A. gueldenstaedtii* might be sustained for various weeks since *tf* was found to be upregulated on day 22 pi in the liver of *A. schrenckii* infected by *M. marinum*^75^. Our results agree with the concept that TRFE behaves as a positive APP in fish, as described for various teleosts (*Puntius sarana, Oreochromis niloticus and Megalobrama amblycephala*) infected by *A. hydrophila*^57,76,77^ or other pathogens^48,57,78,79^. However, this might differ between teleosts, since TRFE has also been described as a negative APP^80,81^. Finally, SAA was the most promising APP among the selected candidates. The *saa* sequence identified in our study matches with only one *saa*-like gene in the recently published genome of *A. ruthenus*^6^, suggesting that as in zebrafish and Atlantic salmon (Ensembl GRCz11, Salmobase) there is only one *saa* gene in sturgeons. This gene was upregulated in the liver during the early bacterial challenge, reaching a tenfold increase at 3 dpc. Moreover, among assayed candidates, it was the unique gene transcriptionally upregulated in a lower inflammatory condition induced by administration of heat-inactivated bacteria (sevenfold at 3 dpc). This finding is novel because there are no reports about positive regulation of hepatic *saa* expression during an acute-phase response in any sturgeon species. Regarding the kinetics of *saa* response in the liver, no changes in *saa* expression were observed after 1 dpc with dead bacteria. Similarly, at 1 dpc, *saa* was not included among the major immune-related genes upregulated in the liver transcriptome of *A. schrenckii* challenged with live *Yersinia ruckeri*^82^. On the other hand, *saa* was found to be upregulated on day 22 pi in the liver of *A. schrenckii* in a well-established *M. marinum* infection^75^. Altogether, upregulation of hepatic *saa* expression in bacterial challenged sturgeons seems to occur on day 1 post-challenge, being detectable at day 3 post-challenge, and sustained during several weeks. The fact that the *saa* response seems to be slower in sturgeon than in mammals may be associated with differences in the thermal physiology between these species. Indeed, endotherm organisms such as mammals exhibited a higher resting metabolic rate than ectotherm fish, reason by which they have an order of magnitude more energy available for physiological functions than does a typical ectotherm of similar mass^83^.

In multiple teleost models of infection, including salmonids, zebrafish, carp and orange-spotted grouper infected by different pathogens (bacteria, fungus, parasites and virus), upregulation of liver *saa* expression indicate that SAA acts as a positive APP^84–87^. These studies demonstrate that *saa* can increase up to 1000 times and its upregulation is sustained for between 7 and 11 days, disappearing when the infection is resolved. Although hepatic SAA response was described at the transcriptional level in these models, no studies examining the correlation between this response and SAA serum levels exist, which may be due to limitations in SAA detection in complex samples such as serum. For instance, SAA was poorly detected by Western blot in serum of rainbow trout infected by *Y. ruckeri*, which was associated with SAA binding to lipid ligands^88^. Besides, an antiserum against a SAA peptide failed to detect SAA by Western blot in the serum, while detecting SAA by immunohistochemistry in the liver and other tissues of infected rainbow trout^89,90^. This antiserum recognizes an 18-residues peptide that does not coincide with p58-*Ag*SAA and p88-*Ag*SAA, which were the *Ag*SAA epitopes detected by the polyclonal antibodies used in our work. On the other hand, SAA levels in serum of infected fish have not been determined by proteomic approaches yet; this analysis likely requires optimizing fractionation protocols since abundant serum proteins could hinder SAA detection. Thus, to examine a putative link between hepatic *saa* expression and serum SAA levels in our sturgeon model, we developed a sandwich ELISA, based on the recognition of p58-*Ag*SAA and p88-*Ag*SAA, which are distant B-cell epitopes on *Ag*SAA 3D-structure and allowed a successful quantification of serum *Ag*SAA. Serum levels of *Ag*SAA increased in both, heat-inactivated and live *A. hydrophila* challenged sturgeons at 3 dpc, and correlated with *saa* upregulation in the liver. Since the comparison of basal levels of *saa* expression in several tissues revealed that hepatocytes are the main source of SAA in Russian sturgeons, the observed correlation would indicate that, as in mammals, *saa* hepatic response had a significant impact on SAA systemic levels during bacterial challenge. Although we cannot rule out the contribution from other organs to circulating *Ag*SAA levels, this would probably be negligible. Since *Ag*SAA upregulation was observed in conditions where non external signs of infection were evident, our results point out that *Ag*SAA is a positive APP and a potential serum biomarker of early infection in Russian sturgeon.

Our results suggest that the acute SAA response is a trait of infection from chondrostean to mammals, contributing to natural defences during bacterial infections. In mammals, in vivo studies support this role for SAA. For instance, in a murine model of *Salmonella typhimurium* infection, *saa*-deficient mice showed a higher bacterial load in the liver and spleen than their wild counterparts did^91^. However, the current understanding of the cellular and molecular mechanisms involved in SAA protective effects is limited. In vitro studies suggest that SAA can bind to Gram-negative bacteria, acting as an opsonin that potentiates phagocytosis^92,93^. This binding ability seems to be shared by SAA from a wide range of species, including fish^36,92,93^. On the other hand, in vitro several effects of SAA on myeloid cells (monocytes and granulocytes), including chemoattraction and induction of pro-inflammatory cytokine secretion were described^28^. Nevertheless, most of these activities might be artefacts caused by the presence of trace levels of bacterial contaminants in the recombinant human SAA preparations used across these studies. Indeed, very recently, most activities of hSAA1 were lost when expressed in eukaryotic cells or depleted from LPS and bacterial lipoproteins, although those associated with binding to formyl peptide receptor 2 might be retained^94,95^.

In conclusion, among several candidates, SAA was the most valuable APP found in Russian sturgeon, highlighting its potential usefulness as a serum biomarker in sturgeon. The existence of additional biomarkers in *A. gueldenstaedtii* should not be discarded since this study employed a biased strategy focused on APP. To investigate this, studies on the liver transcriptome of Russian sturgeons challenged with live bacteria are in progress.

## Materials and methods

### Animals

All animal experiments were performed under strict guidelines of the National Commission of Animal Experimentation (CNEA, Uruguay). Juvenile *Acipenser gueldenstaedtii* (Russian sturgeon, about 1.5-year-old and 300 g in body weight) were generously donated by Esturiones del Río Negro S.A. (Baygorria, Durazno, Uruguay). Sturgeons were transferred to an experimental laboratory at the Instituto de Investigaciones Pesqueras (Facultad de Veterinaria, UdelaR, Uruguay). Fish were randomly distributed in 500 L tanks, supplied with a constant flow rate (1 L/min renewal rate) of fresh, aerated (8 ± 1 mg/L of dissolved oxygen) and de-chlorinated (< 0.2 ppm) tap water (20 ± 2 °C). Fish were fed twice (8:00 am and 16:00 pm) at a feeding rate of 2% fish body mass per day, with a standard pelleted diet formulated and prepared by Esturiones del Río Negro S.A.. Diet composition was 47% protein, 12.5% lipids and 3% fibre (dry matter basis). Fish were acclimated in these conditions at a density of 7 kg/m^3^ for 3 weeks and not fed for 24 h before experimental trials.

### Experimental design and sample collection

All protocols were approved by the Honorary Commission of Animal Experimentation (CHEA, Facultad de Veterinaria, UdelaR, Uruguay). After acclimation, all fish were bled by caudal vein puncture in less than 1 min to minimize handling stress (before treatment samples, BT). For the challenge with dead bacteria, at day 0, two fish groups (n = 11 and 12) were intraperitoneally (ip) injected with 10^9^ CFU/kg body mass of heat-inactivated *A. hydrophila* (a strain isolated from an infection outbreak in an Uruguayan farm, GenBank MF629003). In parallel, two additional fish groups (n = 11) were injected with sterile phosphate buffered saline, pH = 7.2 (PBS). At 1 or 3 dpc, fish from one challenged and one control group were bled as described above, euthanized with eugenol overdose, and dissected to extract liver samples. For live bacteria challenge, fish groups (n = 12) were ip injected with 9.5 × 10^7^ CFU/kg body mass of live *A. hydrophila* or sterile PBS (control) at day 0. At 3 dpc, all fish were bled and then euthanized for liver samples collection. In both experiments, blood samples (2.0 ml) were immediately transferred into tubes to allow clotting at 4 °C. The contracted blood clot was separated from serum by centrifuging at 2800×*g* for 20 min at 4 °C. Fish serum was aliquoted and stored at − 80 °C until use. Serum samples were centrifuged at 10,000×*g* for 20 min at 4 °C prior to use in all assays described below. Furthermore, liver samples (30 mg pieces) were collected under aseptic conditions, placed immediately in RNA later (Qiagen), incubated at 4 °C overnight (ON) and stored at − 80 °C. In parallel, for histopathological analysis, liver pieces (0.5 cm wide) were fixed in Davidson's solution and processed for staining with hematoxylin and eosin^63^. In addition, samples of the spleen, head kidney, brain and gills were collected from unchallenged fish.

### Bioinformatics analysis of *A. sinensis* and *A. baerii* transcriptomic data

At the start of our studies, there were no sturgeon APP sequences available in public DNA repositories or transcriptomic studies involving *A. gueldenstaedtii*. Therefore, to obtain *A. gueldenstaedtii* APP sequences, we first searched for APP homologs in published transcriptomes *of A. baerii*^58^ and *A. sinensis*^9^ two closely related species to *A. gueldenstaedtii.* To that end, all transcriptomes were translated with getorf^96^ and searched with the Blastp algorithm using the protein sequence of the following *D. rerio* APPs as query: HEPC (UniProtKB P61516), ITLN *(NCBI* Reference Sequence XP_021327597.1), SAA (UniProtKB Q642J9), TRFE (UniProtKB A0A2R8RRA6). All bioinformatics analyses were performed using the GALAXY platform^97^. The identity of the putative sturgeon APP homologs found in each transcriptome was confirmed by Blastp against the nr protein database in GenBank. Sequences with the complete coding sequence, highest identity, coverage and e-value were selected. In addition, mined sequences were analysed for conserved protein family domains using InterPro or SMART databases. In the case of HPX/WAP65-2 sequence, the *A. baerii* sequence (unigene GICD01044135.1^61^) was kindly provided by Dr. Denise Vizziano (Facultad de Ciencias, UdelaR, Uruguay).

### Cloning of putative *A. gueldenstaedtii* APPs

APP coding sequences of *A. sinensis* and *A. baerii* were aligned (Clustal Omega^98^, in JalVIew^99^) and specific primers were designed (Supplementary Table S2) to amplify by RT-PCR the corresponding *A. gueldenstaedtii* homologous sequences. In the case of ITLN the primers allowed to amplify the fibrinogen domain, while for the rest APPs the primers were designed to amplify the mature coding sequence. All PCRs were carried out in 50 µl reaction volume containing: 10 µl of 5× High Fidelity buffer, 200 nM dNTPs, 500 nM forward and reverse primer, 25 ng liver and spleen cDNA, 0.01 U Phusion polymerase (Thermo) and molecular biology grade water. PCRs were performed in a Mastercycler thermocycler with specific amplification programs for each APP (Supplementary Table S3). The obtained products (megaprimers) for HEPC (*hamp*)*,* HPX/WAP65-2 (*wap65-2*), ITLN (*itln*)*,* SAA (*saa*) and TRFE (*tf*) were analysed by agarose gel electrophoresis, purified with GeneJET Extraction Kit (Thermo) and quantified in a nanodrop spectrophotometer. Then, they were cloned by RF-cloning^100^ in a modified pET32a vector carrying an ampicillin-resistance cassette^101^ (named p7) by performing a second PCR reaction using 120–200 ng megaprimer, 30 ng p7 vector and the following PCR conditions: a denaturing step at 98 °C for 30 s, 30 amplification cycles (98 °C, for 30 s, 60 °C for 60 s and 72 °C for 5 min), and a final extension step at 72 °C for 7 min. PCR products were then treated with 20 U of DpnI (Thermo) for 2 h at 37 °C to selectively degrade the methylated parental vector. The digestion products were transformed in competent *E. coli* DH5α and *A. gueldenstaedii* APP presence was confirmed by colony-PCR^102^. Positive clones were expanded in LB cultures supplemented with ampicillin (100 µg/ml) and incubated ON at 37 °C. Plasmids were purified using the GeneJET Plasmid Miniprep (Thermo), sequenced (Macrogen) and the *A. gueldenstaedtii* APP sequences deposited in GenBank (Fig. 1d).

### Quantitation by RT-PCR (RT-qPCR) of putative APP expression in the liver

Total liver RNA from *A. gueldenstaedtii* was purified from RNAlater–preserved tissue homogenates using the RNeasy mini kit (Qiagen) according to the manufacturer’s instructions. RNA concentration and purity were determined with a nanodrop spectrophotometer and its integrity confirmed by agarose gel electrophoresis. To synthesize cDNA, 1 µg total RNA was treated with DNAse I (Thermo) and retrotranscribed with M-MLV reverse transcriptase kit (Thermo) following manufacturer’s instructions. RT-qPCR primers for all putative APPs and housekeeping genes (*act-b* and *gapdh*) were designed to work under similar cycling conditions using Primer Express 3.0 and *Acipenser* sequences (Supplementary Table S4). In those cases when more than one sequence was obtained for a putative APP, selected primers were able to amplify all variants. RT-qPCR was carried out in 10 µl reaction volume containing: 5 µl 2X QuantiNova SYBR Green Master Mix (Qiagen), 700–900 nM forward and reverse primers, 2 µl of cDNA (diluted 1:5) and molecular biology grade water, using a Rotor-Gene Q thermocycler (Qiagen). The amplification program was 15 min at 95 °C followed by 40 amplification cycles, each comprised of 15 s at 95 °C followed by 60 s at 60 °C. Amplification of unique RT-qPCR products across samples was verified by melting curve analysis and agarose gel electrophoresis. All primers had amplification efficiencies close to 100%. Relative gene expression across experimental groups was determined with the 2^−ΔΔCT^ method^103,104^ using both, *act-b* and *gapdh* housekeeping genes, which remained constant in all experimental conditions. Absolute quantitation of *saa* mRNA levels in liver, spleen, head kidney, brain and gills from unchallenged juvenile sturgeons were performed by RT-qPCR following the same conditions than that described above. For *saa* mRNA determination, a standard curve (CT vs *saa* mRNA concentration) was built using a *saa* mRNA amplification product as standard.

### Expression and purification of r*Ag*SAA

r*Ag*SAA was overexpressed in *Escherichia coli* BL21 (DE3) Star as a chimeric protein with an amino-terminal His-MBP-tag. Briefly, *saa* cDNA cloned in p7 as described above was sub-cloned in p7-His-MBP and expressed in *E. coli* by induction with 0.5 mM isopropyl-β-D-thiogalactopyranoside (IPTG) at 30 °C during 4 h. Cells were harvested by centrifugation, suspended in lysis buffer (20 mM Tris–HCl pH = 7.2, 500 mM NaCl, 10% w/v glycerol, 0.1% w/v Triton X-100) containing EDTA-free protease inhibitor cocktail (Sigma) and lysed by sonication. The soluble fraction (containing soluble His-MBP-r*Ag*SAA) was separated by centrifugation (20.000×*g* for 1 h at 4 °C) and loaded in a Cu^2+^ immobilized affinity matrix (GE) previously equilibrated with 20 mM Tris–HCl pH = 7.2, 500 mM NaCl, 10% w/v glycerol, 0.05% w/v Tween-20 (equilibration buffer), containing 5 mM imidazole. After 1 h batch incubation at RT, washing and elution steps were carried out with equilibrating buffer containing 10–30 mM imidazole and 300 mM imidazole, respectively. Then, His-MBP-r*Ag*SAA was dialyzed in equilibration buffer, containing 1 mM DTT and digested with His-TEV_SH_ protease^105^. The digested sample was loaded on a new Cu^2+^ immobilized affinity matrix in equilibration buffer containing 50 mM imidazole, and the flow-through (containing r*Ag*SAA and His-MBP as a contaminant) was load on an amylose affinity matrix (NEB) to remove His-MBP. This final step was done twice to ensure complete removal of His-MBP. All r*Ag*SAA purification steps were monitored by Tricine-SDS-PAGE^106^, and protein concentration was determined by Bradford using bovine serum albumin (BSA) as standard^107^. r*Ag*SAA identity was confirmed by MALDI TOF/TOF mass spectrometry analysis of tryptic digested *Ag*SAA and MASCOT searches on a custom database consisting of *A. baerii* and *A. sinensis* translated transcriptomes. r*Ag*SAA oligomeric state was determined by SEC with an AKTA primer plus system using a Superdex 75 10/300 GL column (GE) equilibrated in 20 mM Tris–HCl pH 7.2, NaCl 150 mM, 0.05% v/v Tween-20.

### *Ag*SAA molecular modelling and putative B-cell epitope identification

*Ag*SAA structure was modelled with Swiss Model^108^ using mSAA3 (PDB: 4Q5G.1.A) as the best template (HHblits algorithm^109^). The model was refined with GalaxyRefine^110^ and the lowest qmean structure was selected as the best model^111^. Potential *Ag*SAA B-cell epitopes were identified by alignment analysis (Clustal Omega) using four hSAA A1 peptides (ANYIGSDKYFHARGNYDA, SDARENIQRFFGHG, FFGHGAEDSLADQAANE, GKDPNHFRPAGLPEKY), which are known to be immunogenic^64^. The putative B-cell epitopes were further verified with the Bepipred linear Epitope Prediction algorithm (1.0 version, default settings^112^). To select the most promising *Ag*SAA peptides, their secondary structure was compared against hSAA A1 immunogenic peptides (SAA A1 structure PDB: 4IP9) and their spatial orientation was studied in the *Ag*SAA modelled structure (PyMOL Molecular Graphics System Version 2.0 Schrödinger, LLC).

### Polyclonal anti-*Ag*SAA antibodies

Polyclonal antibodies against two putative *Ag*SAA B-cell epitopes (SNGREAWQSFRGSG, GGDPNDYRPAGLPSKY, named p58-*Ag*SAA and p88-*Ag*SAA respectively) conjugated to keyhole limpet hemocyanin (KLH, GenScript) were raised in rabbits. Animal handling, inoculation and sampling were performed following a protocol approved by CHEA (Facultad de Química, UdelaR, Uruguay). Briefly, rabbits were inoculated at four separate subcutaneous sites with 400 µg of peptide-KLH conjugated emulsified in Freund’s complete adjuvant (Sigma). Antibody response was monitored by collecting blood samples every 2–3 weeks and measuring antiserum titles and avidity by ELISA coated with r*Ag*SAA and following conventional protocols^113,114^. Antibodies raised against *Ag*SAA peptides were purified by affinity chromatography on a r*Ag*SAA-Sepharose matrix prepared using N-hydroxysuccinimide activated agarose matrix (GE) following manufacture´s protocol. Then, antiserum containing anti-*Ag*SAA peptide antibodies was diluted in TBS buffer (20 mM Tris–HCl pH 7.4, 150 mM NaCl) and applied to r*Ag*SAA-NHS agarose matrix previously equilibrated in TBS buffer. The matrix was extensively washed with TBS buffer and 20 mM Tris–HCl pH 7.4, 500 mM NaCl, 0.2% w/v Tween-20. Antibodies against *Ag*SAA peptides were eluted with 20 mM Glycine–HCl pH = 2.0, 150 mM NaCl and immediately neutralized. Purified antibodies were dialyzed ON at 4 °C against PBS, concentrated with 10 kDa centrifugal filters (Pall) and stored at − 20 °C. Purified anti-p88-*Ag*SAA antibodies were biotinylated using EZ-link Sulfo-NHS-LC-biotin (Thermo) and following manufacturer´s protocol. Antibody reactivity and purification steps were assessed by Western Blot.

### Western blotting

Samples were analysed by SDS-PAGE^115^, using prestained protein Mw marker (Thermo), and then blotted ON to a PVDF membrane (Millipore) using a Mini Protean Tetra Cell and Blot Module (Bio-Rad) according to manufacturer’s instructions. Membranes were blocked with PBS containing 0.05% w/v Tween-20 and 0.2% w/v BSA (blocking buffer) for 1 h at RT. Afterwards, membranes were incubated with primary rabbit polyclonal antibodies for 1 h at RT and then washed 4 times with PBS containing 0.05% w/v Tween-20 (PBS-T). For detection, membranes were incubated with goat anti-rabbit IgG conjugated to peroxidase (Calbiochem, diluted 1:2000 in blocking buffer) for 1 h at RT. Membranes were extensively washed and developed with ECL SuperSignal West Pico Plus Chemiluminescent substrate (Thermo) using Gbox (Syngene).

### ELISA for *Ag*SAA quantitation

A sandwich ELISA was developed to quantify *Ag*SAA in serum samples using anti-p58-*Ag*SAA as capture antibody, biotinylated anti-p88-*Ag*SAA as detection antibody and r*Ag*SAA as standard. Nunc 96-well plates (Thermo) were coated ON with 0.1 µg/well anti-p58-*Ag*SAA, blocked for 1 h at RT (PBS, 0.5% w/v BSA) and washed. All washing steps were carried out 4 times with PBS containing 0.05% w/v Tween-20 for 5 min each, at RT. Plates were then incubated for 1 h at 37 °C with 100 µl/well of r*Ag*SAA (serial dilutions from 500 to 0.69 ng/ml, in duplicates) or *A. gueldenstaedtii* serum, both diluted in binding buffer (PBS, 0.5% w/v BSA, 0.05% w/v Tween-20). After washing, *Ag*SAA bound to rabbit immunoglobulins was detected by incubation (1 h at 37 °C) with 100 µl/well of biotinylated anti-p88-*Ag*SAA (0.152 µg/ml in binding buffer) followed by washing steps and incubation (45 min at 37 °C) with 100 µl/well of streptavidin-peroxidase (Sigma, 0.1 µg/ml in binding buffer). Plates were then washed and the peroxidase activity developed by adding 100 µl/well of TMB peroxidase substrate solution (50 mM citric acid, 105 mM Na_2_HPO_4_, 100 µg/ml of 3,3 ´,5,5 ´- tetramethylbenzidine and 0.006% v/v H_2_O_2_) and incubating 15 min at RT. The enzyme reaction was stopped by addition of 50 µl 0.3 M H_2_SO_4_. Absorbance was read at 450 nm (A_450nm)_ using a 96-well plate spectrophotometer (Multiskan MS Labsystems). Data were analysed using the Origin 8.5 software (OriginLab, Northampton, MA). The calibration curve (A_450nm_ vs r*Ag*SAA) was fitted to a sigmoidal function of four parameters: Y = Amax_450nm_ + (Amin_450nm_- Amax_450nm_)/(1 + (x/IC50)^n) were Amax_450nm_ and Amin_450nm_ correspond to the maximum and minimum absorbances at the theoretical infinite and zero concentrations, respectively; IC50 is the mid-range concentration and n is the slope factor. The minimal *Ag*SAA concentration detected in binding buffer was calculated by interpolating the mean of blanks plus three standard deviations on the calibration curve. However, *Ag*SAA detection in serum samples might be affected by the presence of serum components (matrix effect). This effect was assessed using a pool of Russian sturgeon sera from four acclimated and untreated fish, showing no detectable *Ag*SAA by Western Blot. Dilutions (between 1:5 and 1:500 in PBS) of this pool were prepared, spiked with r*Ag*SAA (6.2, 55.6, 166.7 and 500 ng/ml) and the percentage of recovered r*Ag*SAA was determined by the developed ELISA. As shown in Supplementary Fig. S10b, 1:200 was the minimal serum dilution at which the serum matrix effect was overcome. *Ag*SAA concentration in serum samples was then determined using various serum dilutions, from 1:200 to 1:10,000, to avoid interpolation at the extremes of the curve where the relative error was high (≥ 15%, Supplementary Figure S10b).

### Statistical analysis

Statistical analysis was performed using Graphpad Prism 6 (Graphpad, La Jolla, Ca). The bulk of data corresponding to liver APP mRNA expression and *Ag*SAA serum concentrations did not show Gaussian distribution. Therefore, for intergroup comparison at different time points (i.e. before vs post-treatment) or between treatments (i.e. PBS vs *A. hydrophila*, at each time point), data was transformed using the inverse square root or logarithm to meet normality (verified using D’ Agostino-Pearson omnibus normality test). Afterwards, comparisons were performed using the unpaired Student's t-test with Welch's correction or one-way analysis of variance (ANOVA) with post-hoc Tukey´s multiple comparison test, as appropriate (p < 0.05). Correlation between *Ag*SAA serum levels and relative liver *saa* mRNA levels was analysed using the non-parametric, two-tailed Spearman test (*p* < 0.05).

## Supplementary Information

Supplementary Information
