# Supplementary material for: Serum amyloid A is a positive acute phase protein in Russian sturgeon challenged with Aeromonas hydrophila
Source: Sci Rep. 2020 Dec 17;10:22162. doi: 10.1038/s41598-020-79065-9 (PMC7746741; doi:10.1038/s41598-020-79065-9)
Supplement: Supplementary file 1 — Supplementary Information [file 41598_2020_79065_MOESM1_ESM.pdf]

Supplementary Figures

Serum Amyloid A is a positive Acute Phase Protein in Russian sturgeon challenged with *Aeromonas hydrophila*

Mauricio Castellano[1,3], Valeria Silva-Álvarez [1], Marcio Aversa[1], María Lamas-Bervejillo [1], Ignacio Quartiani[2], Alejandro Perretta[2], Andrea Villarino[3,*] and Ana María Ferreira[1,*].

[1]Cátedra de Inmunología, Facultad de Ciencias, Facultad de Química, Universidad de la República, CP 11600, Montevideo, Uruguay.

[2]Instituto de Investigaciones Pesqueras, Facultad de Veterinaria, Universidad de la República, CP 11300, Montevideo, Uruguay.

[3]Sección Bioquímica y Biología Molecular, Facultad de Ciencias, Universidad de la República, CP 11400, Montevideo, Uruguay.

[*]Correspondence and requests for materials should be addressed to A.V. (avillarino@fcien.edu.uy) and A.M.F (aferreira@fcien.edu.uy).

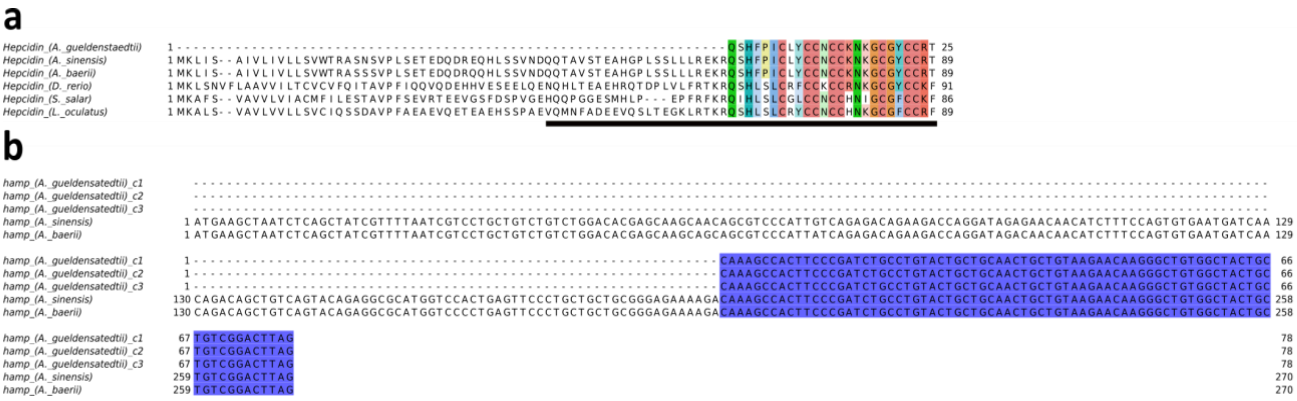

Figure S1. Teleost and sturgeon HEPC alignment. (a) Amino acid sequence alignment of mature HEPC from *A. gueldenstaedtii* (Russian sturgeon, consensus sequence), *A. sinensis* (Chinese sturgeon, translated using GenBank ID GETX01012667.1), *A. baerii* (Siberian sturgeon, translated from transcript comp130481_c0_seq1,[58]), *D. rerio* (Zebrafish, UniProtKB P61516.1), *S. salar*, (Atlantic salmon, UniProtKB Q801Y3.1) and *L. oculatus* (Spotted Gar, translated using GenBank ID XP_006641712.1), using Clustal Omega. The Pfam domain 06446 corresponding to HEPC is underlined in black. **(b)** Nucleotide alignment of *hamp* from *A. gueldenstaedtii* (three identical clones, GenBank ID MN544258) with the entire *hamp* coding sequences from *A. sinensis* (GenBank ID GETX01012667.1) and *A. baerii* (transcript comp130481_c0_seq1,[58]), using Clustal Omega. The sequence identity is represented in shades of blue.

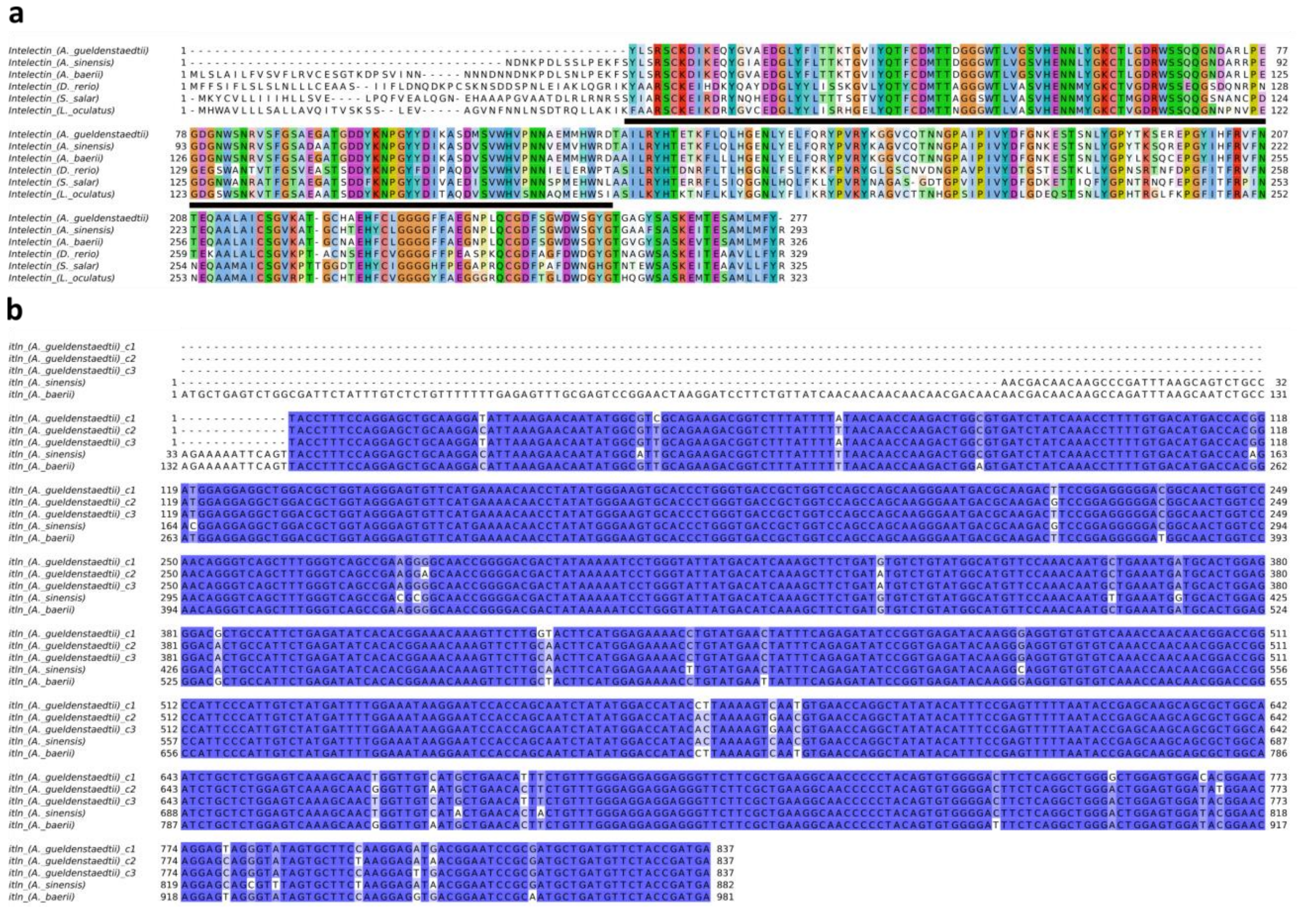

Figure S2. Teleost and sturgeon ITLN alignment. (a) Amino acid sequence alignment of the fibrinogen domain of ITLN from *A. gueldenstaedtii* (Russian sturgeon, consensus sequence), *A. sinensis* (Chinese sturgeon, translated using GenBank ID GETX01010297.1), *A. baerii* (Siberian sturgeon, translated from transcript comp135402_c0_seq1,[58]), *D. rerio* (Zebrafish, translated from GenBank ID XP_021327597.1), *S. salar*, (Atlantic salmon, UniProtKB A0A1S3MFB1) and *L. oculatus* (Spotted Gar, translated from GenBank ID XP_006641047.1) using Clustal Omega. The SM00186 fibrinogen related domain is shown in black. **(b)** Nucleotide alignment of the fibrinogen domain *itln* sequences from *A. gueldenstaedtii* (clones c1-c3, GenBanK ID: MN544255, MN544256, MN544257), *A. sinensis* (GenBank ID GETX01010297.1) and *A. baerii* (translated from transcript comp135402_c0_seq1,[58]), using Clustal Omega. The sequence identity is represented in shades of blue.

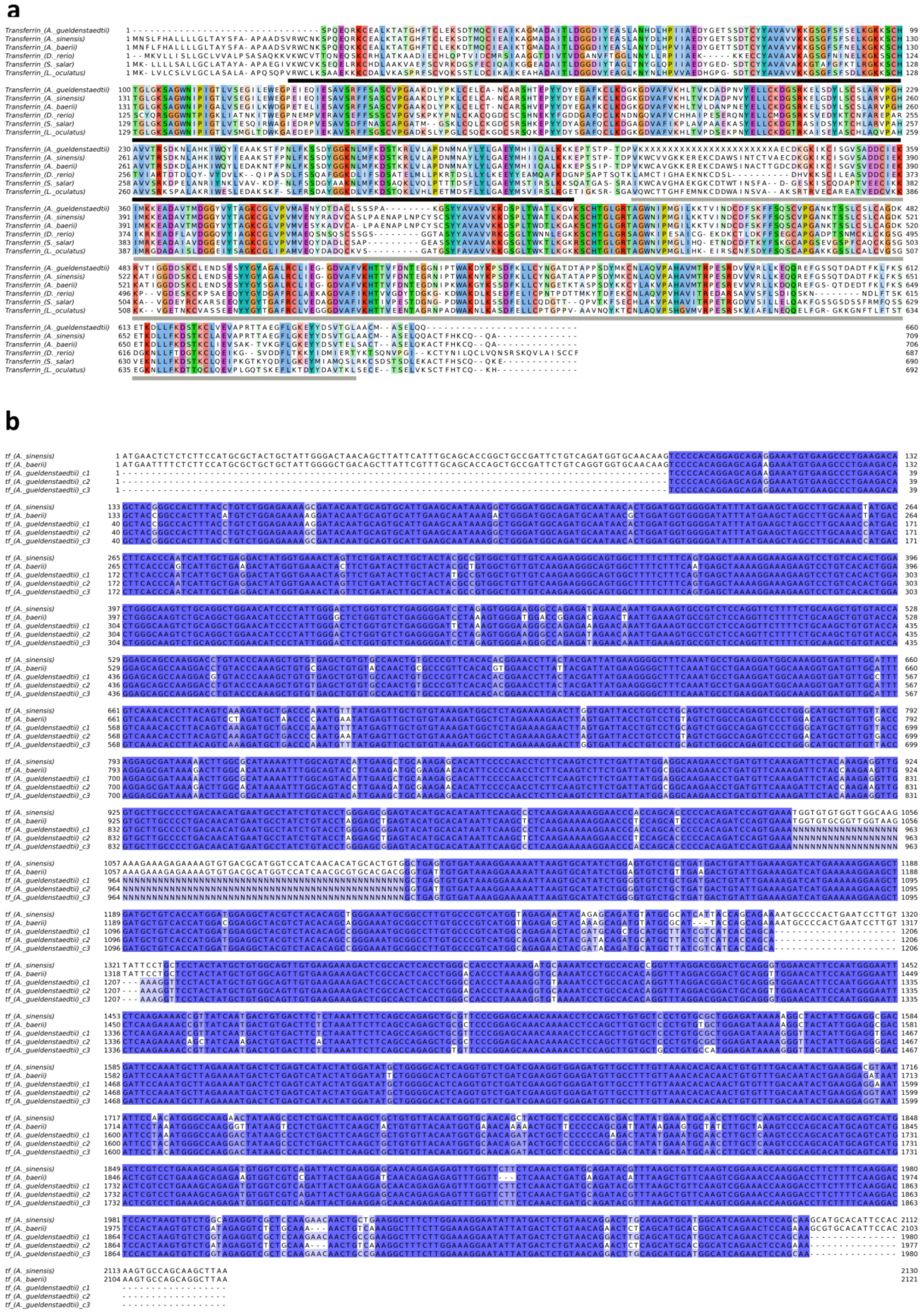
a
Transferrin_(A._gueldenstaedtii)
Transferrin_(A._sinensis)
Transferrin_(A._baerii)
Transferrin_(D._rerio)
Transferrin_(S._salar)
Transferrin_(L._oculatus)
b
tf_(A._sinensis)
tf_(A._baerii)
tf_(A._gueldenstaedtii)_c1
tf_(A._gueldenstaedtii)_c2
tf_(A._gueldenstaedtii)_c3

Figure S3. Teleost and sturgeon TRFE alignment. (a) Amino acid sequence alignment of mature TRFE from *A. gueldenstaedtii* (Russian sturgeon, partial consensus sequence), *A. sinensis* (Chinese sturgeon, translated using GenBank ID GETX01026229.1), *A. baerii* (Siberian sturgeon, translated from transcript comp137559_c1_seq1,[58]), *D. rerio* (Zebrafish, UniProtKB A0A2R8RRA6), *S. salar*, (Atlantic salmon, GenBank ID UniprotKB P80426) and *L. oculatus* (Spotted Gar, UniProtKB W5MU77), using Clustal Omega. The Pfam domain 00405 and the sequence between amino acids residues 345 and 686 corresponding to TRFE, are shown in black and gray, respectively. **(b)** Nucleotide sequence alignment of mature *tf* sequences from *A. gueldenstaedtii* (clones c1-c3: MT215069, MT215070, MT215071), *A. sinensis* (GenBank ID GETX01026229.1) and *A. baerii* (transcript comp137559_c1_seq1,[58]) using Clustal Omega. The sequence identity is represented in shades of blue.

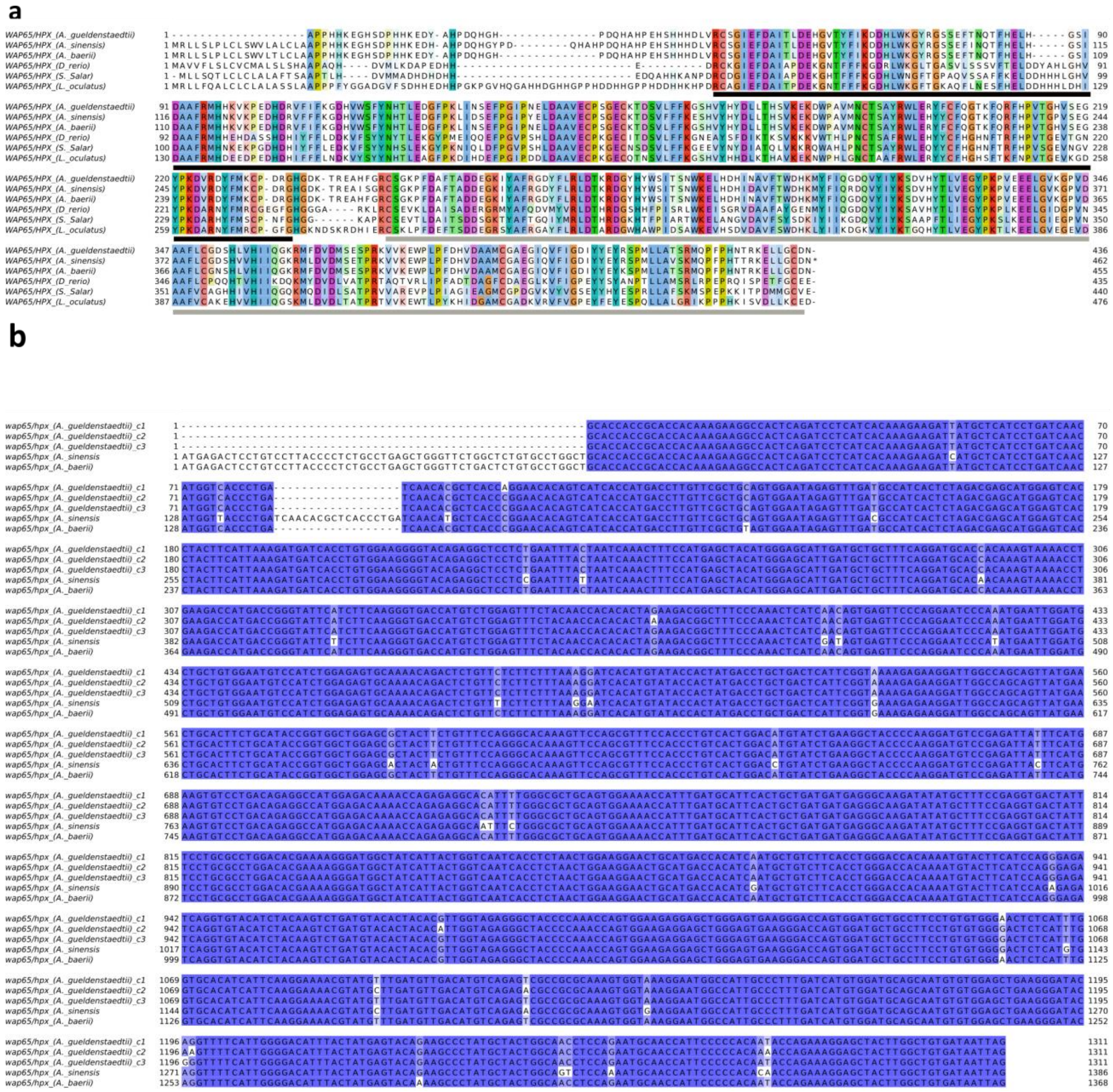

Figure S4. Teleost and sturgeon HPX/WAP65-2 alignment. (a) Amino acid sequence alignment of mature HPX/WAP65-2 from *A. gueldenstaedtii* (Russian sturgeon, consensus sequence), *A. sinensis* (Chinese sturgeon, translated using GenBank ID GETX01015919.1), *A. baerii* (Siberian sturgeon, translated from transcript GICD01044135.1[61], *D. rerio* (Zebrafish, translated from GenBank ID XP_005173505.1, corresponding to Hemopexin 2 according to UniProtKB E7FFU0), *S. salar* (Atlantic salmon, UniProtKB A0A1S3MVQ2) and *L. oculatus* (Spotted Gar, translated from GenBank ID XP_015219463.1). The two hemopexin-like domains, IPR036375 and SSF50923 are shown in black and gray, respectively. **(b)** Nucleotide alignment of mature *hpx/wap65-2* sequences from *A. gueldenstaedtii* (clones c1-c3: MN544252, MN544253, MN544254), *A. sinensis* (GenBank ID GETX01015919.1) and *A. baerii* (transcript ID GICD01044135[61]) using Clustal Omega. The sequence identity is represented in shades of blue.

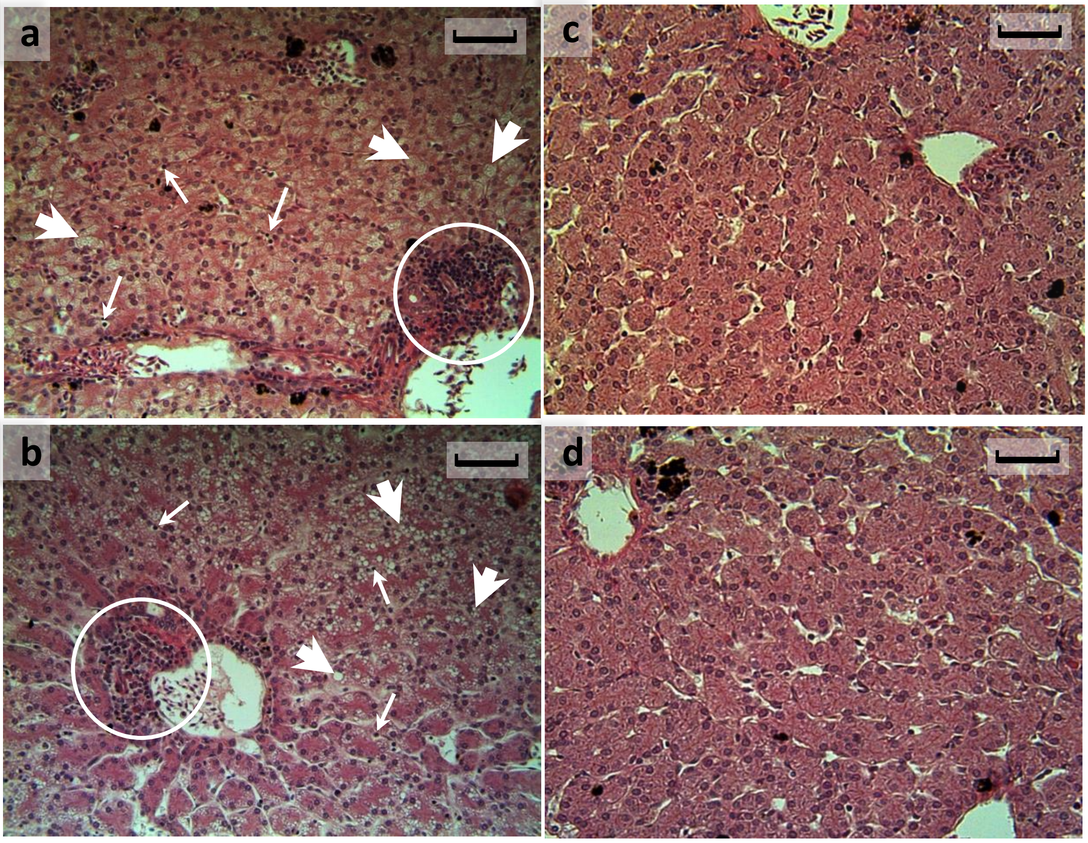

Figure S5. Histological liver analysis of Russian sturgeon challenged with *A. hydrophila*. Sturgeons were ip injected with heat-inactivated **(a)** or live **(b)** *A. hydrophila* or PBS as control (**c** and **d**). Small liver pieces were fixed in Davidson's solution and processed for staining with hematoxylin and eosin. Leukocyte infiltrates in portal space are indicated by white circles, while illustrative pyknotic nuclei and hepatocyte vesiculation are indicated with small and big white arrows, respectively. Images are representative of 11 and 7 challenged or control fish, respectively. Bars correspond to 100 μm.

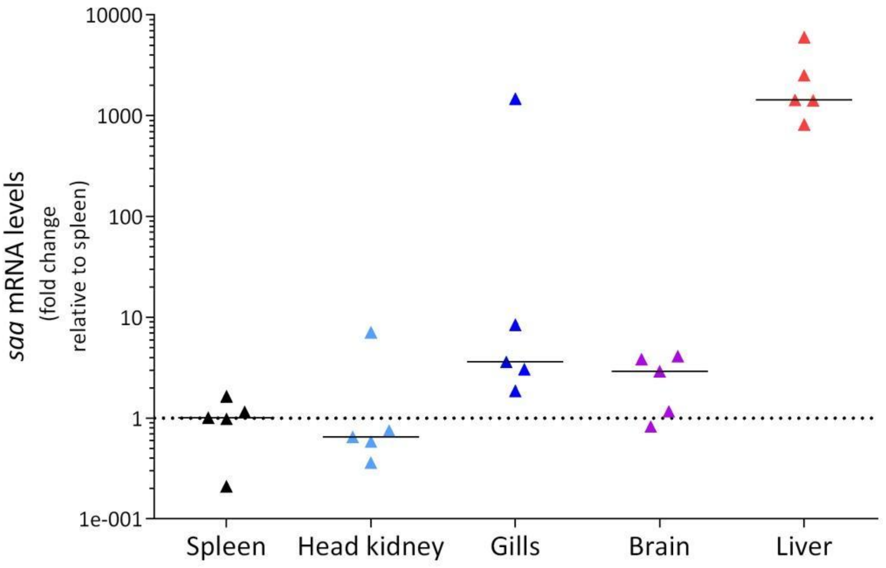

Figure S6. **Analysis of *saa* mRNA expression in spleen, head kidney, gills, brain and liver in Russian sturgeons.** mRNA was obtained from tissue samples from five juvenile sturgeons acclimated for 24 days as described in the Methods Section. Absolute *saa* mRNA levels were quantified by RT-qPCR. The graph shows the *saa* mRNA level expressed relative to spleen level (fold change).

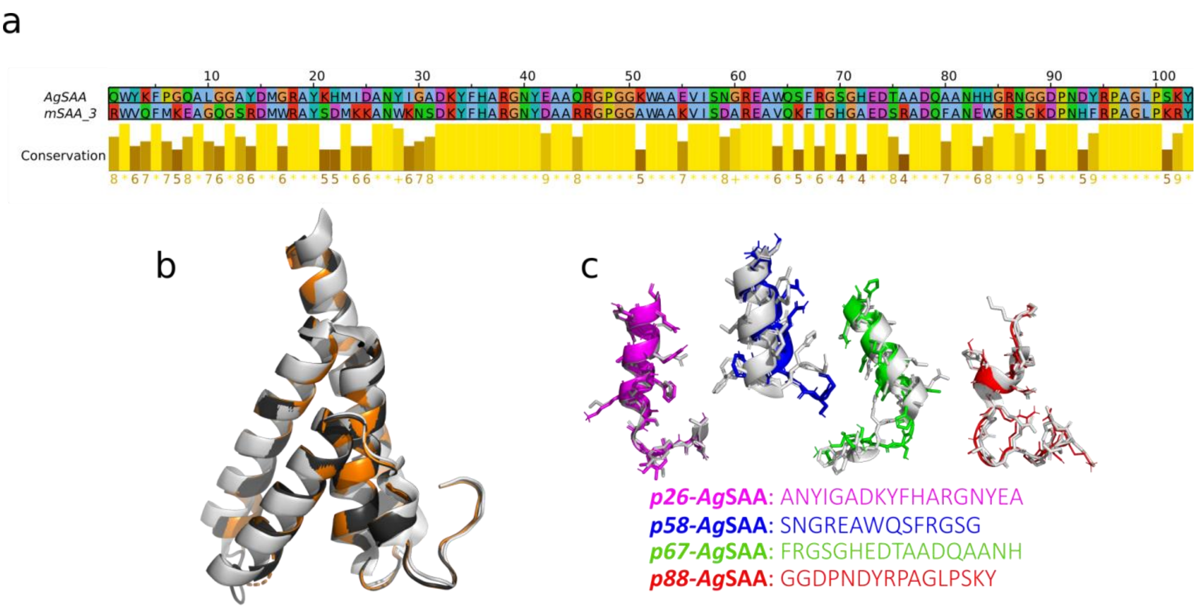

Figure S7. Structural alignment of *Ag*SAA with *h*SAA1, *m*SAA3 and potential immunogenic SAA peptides. (a) Amino acid sequence alignment of mature *Ag*SAA with mature *Mus musculus* SAA3 (*m*SAA3) using Clustal Omega. Yellow bar plots highlight their aminoacidic conservations. **(b)** Structural alignment of *Ag*SAA model (black) with *h*SAA1 structure (PDB: 4IP9, gray) and *m*SAA3 (PDB: 4Q5G, orange) using PyMOL. **(c)** Structural alignment of peptides 1, 2, 3 and 4 of *h*SAA (gray) with p26-*Ag*SAA (magenta), p58-*Ag*SAA (blue), p67-*Ag*SAA (green) and p88-*Ag*SAA (red). The sequences corresponding to *Ag*SAA peptides are shown.

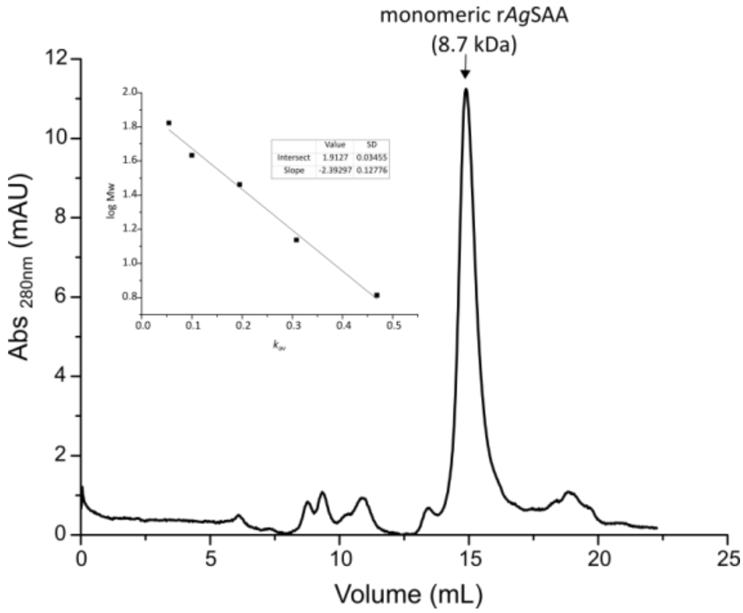

Figure S8. Analysis of the oligomerization state of r*Ag*SAA. Size exclusion chromatography of r*Ag*SAA (150 µg) in a Superdex 75 10/300 GL matrix equilibrated in 20 mM Tris-HCl pH 7.2, NaCl 150 mM, 0.05% v/v

Tween20, and run at a flow rate of 0.4 mL/min. Monomeric r*Ag*SAA (theoretical Mw 11.4 kDa) was eluted at 14.9 mL, corresponding to an apparent Mw of 8.7 kDa. Inset shows the calibration curve of the Superdex analytical column, obtained using SEC Mw markets (Sigma).

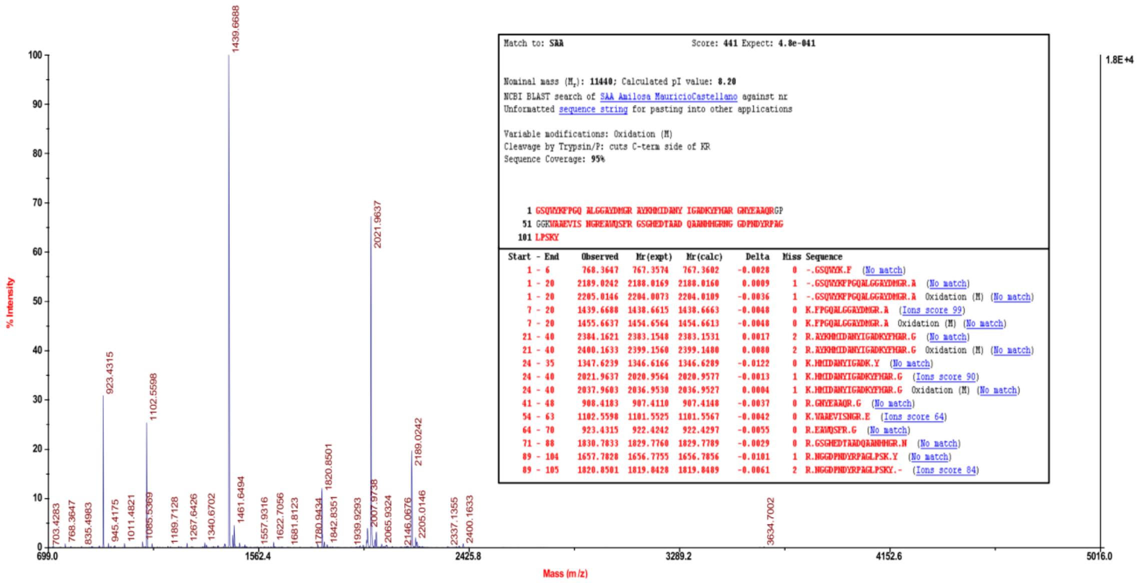

Figure S9. Identity analysis of r*Ag*SAA by MALDI-TOF/TOF MS. Purified r*Ag*SAA was digested with trypsin (sequence grade, Promega), desalted using C18 reverse phase micro-columns (OmixHTips, Varian) and eluted directly onto the MALDI sample plate with matrix solution a-cyano-4-hydroxycinnamic acid in 60% ACN containing 0.1% TFA. Mass spectra of peptides mixtures were acquired in a 4800 MALDI TOF/TOF instrument (ABi Sciex) in positive reflector mode, and externally calibrated using a mixture of peptide standards (Applied Biosystems). Proteins were identified with measured m/z values using the MASCOT software (Matrix Science, http://www.matrixscience.com) and an in-house Acipenser database, which was built translating all sequences of *A. baerii* and *A. sinensis* transcriptomes[58,9]. The identity of r*Ag*SAA was confirmed with 95% sequence coverage.

a

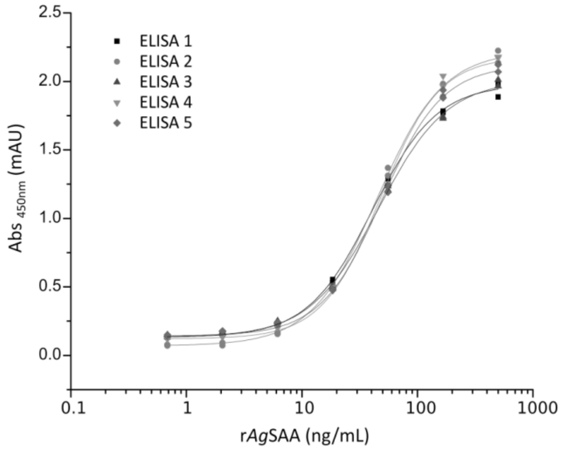

b

		Russian Sturgeon Serum							
		1/20 dilution		1/100 dilution		1/200 dilution		1/500 dilution	
**Spiked level**	Expected [SAA]	Observed [SAA]	Recovery (%)	Observed [SAA]	Recovery (%)	Observed [SAA]	Recovery (%)	Observed [SAA]	Recovery (%)
**Low (6.2 ng/mL)**	6.72 ± 0.03	2.75 ± 0.3	41 ± 5	5.5 ± 0.3	82 ± 5	6.4 ± 0.1	95 ± 1	6.5 ± 0.1	97 ± 6
**Med (55.6 ng/mL)**	54.9 ± 0.1	49.7 ± 2	90 ± 4	56 ± 2	101 ± 4	57 ± 3	104 ± 5	57 ± 2	104 ± 3
**High (166.7 ng/mL)**	179 ± 2	165 ± 2	92 ± 1	165 ± 3	92 ± 2	170 ± 10	95 ± 6	166 ± 3	92 ± 2
**Very high (500 ng/mL)**	497 ± 52	415 ± 77	83 ± 16	437 ± 73	88 ± 15	501 ± 87	101 ± 17	479 ± 83	96 ± 16

Figure S10. Development of a sandwich ELISA for serum *Ag*SAA. (a) The sandwich ELISA was developed using anti-p58-AgSAA-Ig as capture antibody, biotinylated anti-p88-AgSAA-Ig as detection antibody and r*Ag*SAA as standard. The graph shows the calibration curves obtained using purified r*Ag*SAA (0.69 – 500 ng/mL, in duplicates) in five independent assays. Plotted Abs450nm values were calculated by subtracting the mean of blanks wells. The curve was fitted to a sigmoidal function of four parameters as described in Material and Methods. **(b)** Matrix effect or Russian sturgeon´s serum. The table shows the percentage of r*Ag*SAA recovered when it was spiked at four different levels (low, medium, high and very high) in different dilutions of a pool of Russian sturgeon sera. Data show that 1:200 was the minimal serum dilution at which the serum matrix effect was overcome.

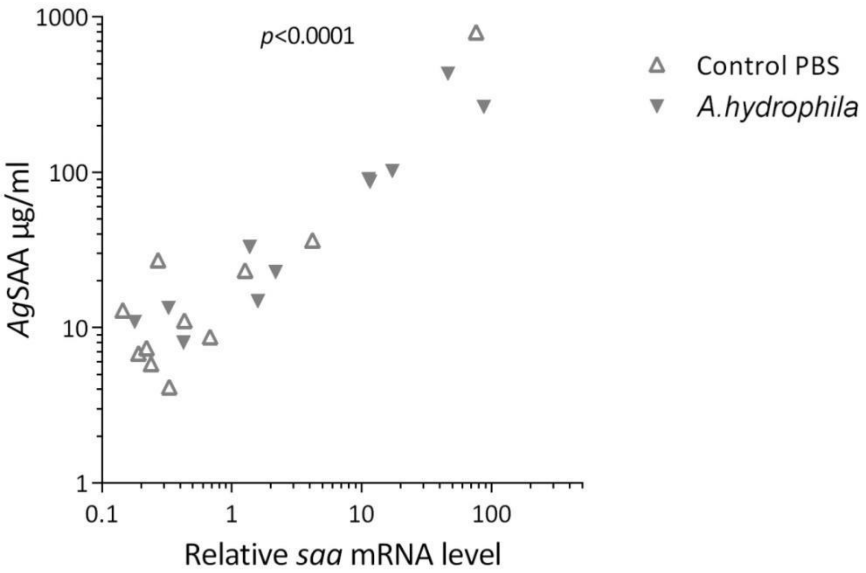

Figure S11. Correlation between *Ag*SAA serum levels and relative *saa* mRNA levels in sturgeons challenged with heat-inactivated *A. hydrophila*. Juvenile Russian sturgeons were ip challenged with heat-inactivated *A. hydrophila* or sterile PBS (control PBS), and liver APP expression analyzed at 3 dpc. Liver *saa* mRNA levels were determined by qPCR (using the $2^{-\Delta\Delta CT}$ method and *gapdh* as housekeeping gene), and results expressed as the increase (fold change) relative to the corresponding control group. *Ag*SAA serum levels were determined by sandwich ELISA. Graph shows the correlation between *saa* mRNA expression in liver and *Ag*SAA serum levels. This correlation was also observed when *saa* mRNA levels were determined by qPCR using *act-b* as housekeeping gene.

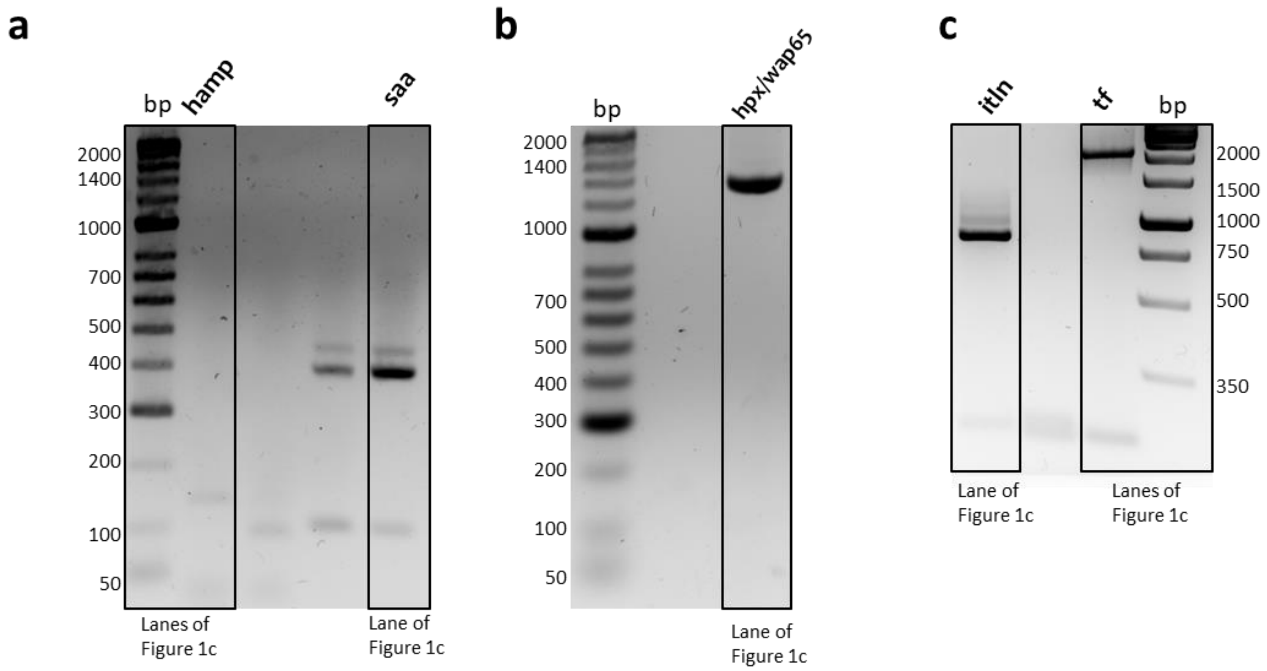

Figure S12. Original agarose gels showing RT-PCR products corresponding to *A. gueldenstaedtii amplicons:* *hamp,* saa **(a)**, *hpx/wap65* **(b)**, *itln* and *tf* **(c)**. The region of interest in each gel used to compose Figure 1c is shown with a black box.

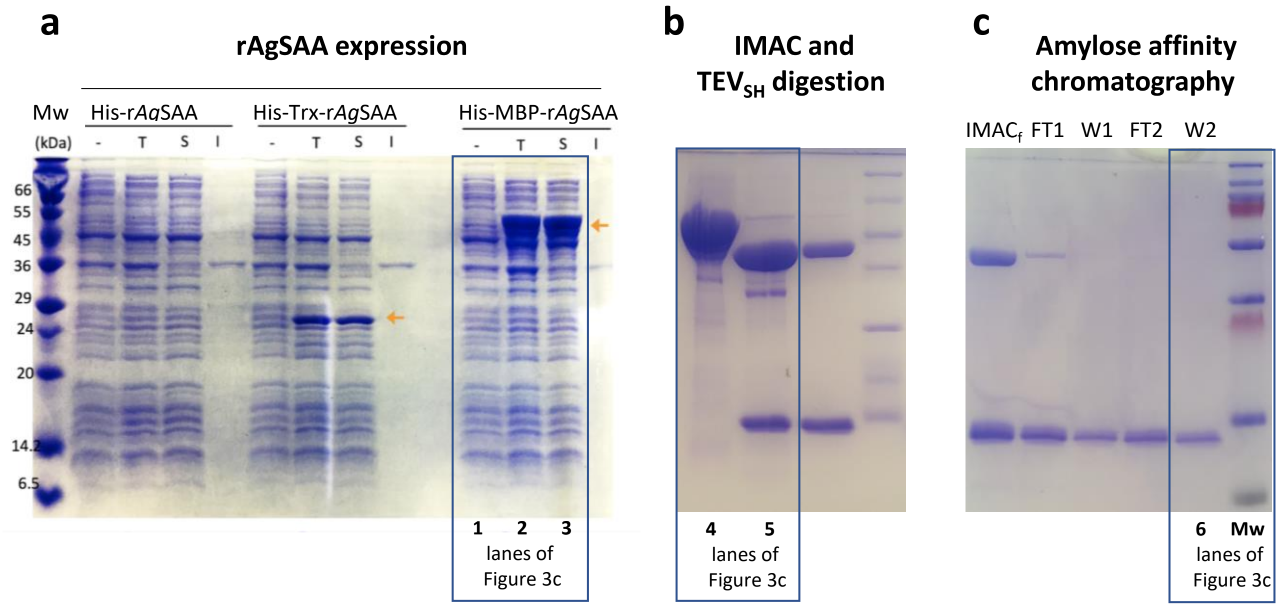

Figure S13. *E. coli* expression and purification of r*Ag*SAA. a) Coomassie-stained SDS-PAGE-TRICINE analysis of the *E. coli* expression of r*Ag*SAA with different N-terminal tags. Samples corresponding to the total fraction of uninduced cultures (-) and the total (T), soluble (S) and insoluble (I) fractions of the IPTG-induced cultures of His-r*Ag*SAA, His-Thioredoxin-r*Ag*SAA and His-MBP-r*Ag*SAA are shown. The region of interest in this gel used to compose Figure 3c (lanes 1 to 3) is shown with a blue box. **b)** Coomassie-stained SDS-PAGE-TRICINE analysis of fractions obtained by IMAC of His-MBP-r*Ag*SAA, corresponding to the elution of the IMAC and TEV-digestion of His-MBP-*Ag*SAA (lanes 4 and 5 of Figure 3c, respectively). The region of interest in this gel used to compose Figure 3c is shown with a blue box. **c)** Coomassie-stained SDS-PAGE-TRICINE analysis corresponding to the purification of r*Ag*SAA by amylose affinity chromatography. The figure shows the analysis of the TEV-digested His-MBP-*Ag*SAA fraction obtained by IMAC (lane IMAC$_f$) as well as the flow through (FT1 and FT2) and washing fractions (W1 and W2) obtained by two sequential purification steps. The region of interest in this gel used to compose Figure 3c (lane 6 and MW) is shown with a blue box.

Table S1. Identification of *A. sinensis* and *A. baerii* putative APPs in available transcriptomic datasets. *A. sinensis*[9] and *A. baerii* transcriptomes[58] were searched for sequences with homology to selected APP using the blastp algorithm and *Danio rerio* APP as queries. In the case of HPX/WAP65 the search was only performed in *A. sinensis* transcriptome since *A.baerii* sequence was provided by Dr. D. Vizziano (unigene GICD01044135.1[61]). Results correspond to the first hit and indicate the unigene access code of the longest transcript, the identity, and the e-value with regard to the *Danio rerio* sequence employed for each search. To confirm their identity, the putative APP sturgeon sequences found were analyzed by blastp against the NCBI nr database. For each of these blasts, the sequence description, accession, identity and e-value corresponding to the first hit is shown. Putative sturgeon APPs were searched for conserved protein domains (CPD) using the InterPro database.

**APPs** (*Danio rerio*)	**Unigene**	**Identity (%)**	**E-value**	**Description**	**Accession**	**Identity (%)**	**E-value**	**Acipenser CPD**
	**BLASTp against *A. sinensis* transcriptome**				**BLASTp against NCBI nr database**			**InterPro database**
**HEPC**	GETX01012667.1	36	5.65E-04	**HEPC**	XP_006641712.1	46	2.00E-13	Pfam 06446
**HPX/WAP65**	GETX01015919.1	49	1.24E-153	**HPX/WAP65**	XP_015219463.1	57	0	IPR036375, SSF50923
**ITLN**	GETX01010297.1	67	4.40E-128	**ITLN**	XP_015222783.1	73	3.00E-132	Smart SM00186
**SAA**	GETX01015093.1	69	1.65E-49	**SAA**	CEG62717.1	72	4.00E-53	Pfam 00277
**TRFE**	GETX01026229.1	56	0	**TRFE**	XP_015217073.1	59	0	Pfam 00405
	**BLASTp against *A. baerii* transcriptome**				**BLASTp against NCBI nr database**			**InterPro database**
**HEPC**	comp130481_c0_seq1	36	5.65E-04	**HEPC**	XP_006641712.1	46	2.00E-13	Pfam 06446
**ITLN**	comp135402_c0_seq1	63	5.42E-145	**ITLN**	XP_015222783.1	63	3.00E-155	Smart SM00186
**SAA**	comp122687_c0_seq1	69	5.06E-55	**SAA**	CEG62717.1	70	6.00E-52	Pfam 00277
**TRFE**	comp137559_c1_seq1	57	0	**TRFE**	XP_015217073.1	59	0	Pfam 00405

Table S2. Primers designed to amplify and clone *A. gueldenstaedtii* putative APPs. Primers were designed for RF-cloning[100], thus non-underlined and underlined sequences were complementary to the p7 vector and target gene, respectively. For primer design the coding sequence of *hamp, hpx/wap65*, *itln* fibrinogen domain (SMART code: SM00186)*, saa and tf* were used.

Gene	Forward primer	Reverse primer
hamp	5’-GGATCGGAAAACCTGTATTTTCAGGGATCC CAAAGCCACTTCCCGATCTGC-3’	5’-GAACTGCGGGTGGCTCCAGCTGCCGGATCC CTAAGTCCGACAGCAGTAGCCA-3’
hpx/wap65	5’-GGATCGGAAAACCTGTATTTTCAGGGATCC5’-GGATCGGAAAACCTGTATTTTCAGGGATCC GCACCACCGCACCACAAA-3’	5’-GAACTGCGGTGGCTCCAGCTGCCGGATCCC5’-GAACTGCGGGTGGCTCCAGCTGCCGGATCCC TAATTATCACAGCCAAGTAGCTCC-3’
itln	5’-GGATCGGAAAACCTGTATTTTCAGGGATCC5’-GGATCGGAAAACCTGTATTTTCAGGGATCC TACCTTTCCAGGAGCTGCA-3’	5’-GAACTGCGGGTGGCTCCAGCTGCCGGATCC5’-GAACTGCGGGTGGCTCCAGCTGCCGGATCC TCATCGGTAGAACATCAGCATC-3’
saa	5’-GGATCGGAAAACCTGTATTTTCAGGGATCC5’-GGATCGGAAAACCTGTATTTTCAGGGATCC CAATGGTATAAGTTCCCAGGCC-3’	5’-GAACTGCGGGTGGCTCCAGCTGCCGGATCC5’-GAACTGCGGGTGGCTCCAGCTGCCGGATCC TCAGTATTTCGAAGGGAGGCC-3’
tf	5’-GGATCGGAAAACCTGTATTTTCAGGGATCC5’-GGATCGGAAAACCTGTATTTTCAGGGATCC GCACCAGCTGCCGATTCT-3’	5’GAACTGCGGGTGGCTCCAGCTGCCGGATC5’-GAACTGCGGGTGGCTCCAGCTGCCGGATCCTT AAGCTTGCTGGCACTTGT-3’

Table S3. PCR program to amplify *A. gueldenstaedtii* putative APPs from cDNA

Gene	Initial DNA denaturation	Amplification cycle			Final extension
		Denaturation	Annealing	Extension	
hamp	98°C/30 s	98°C/8 s	58°C/20s	72°C/21 s	72°C/ 5 min
hpx/wap65			58°C/20s	72°C/45 s	
itln			57°C/20s	72°C/30 s	
saa			58°C/20 s	72°C/21 s	
tf			58°C/20 s	72°C/60 s	

Table S4. Primers designed to study *A. gueldenstaedtii* APPs gene expression by RT-qPCR

Gene	Forward primer	Reverse primer
actb *(housekeeping)*	5'-ACACCCCAGCCATGTACGTT-3'	5'-ACACCATCACCAGAGTCCATCA-3'
gapdh *(housekeeping)*	5'-GCCTACACAGCCACACAGAAGA-3'	5'-GGTGCTGGCTGGAATGATGT-3'
hamp	5'-CTGCGGGAGAAAAGACAAAGC-3'	5'-AGCCCTTGTTCTTACAGCAGTTG-3'
hpx/wap65	5'-GGTGCACATCATTCAAGGAAAA-3'	5'-CACATGATCAAAGGGCAATGG-3'
itln	5'-GGTGAGATACAAGGGAGGTGTGT-3'	5'-TGCTGGTGGATTCCTTATTTCC-3'
saa	5'- ATCGGTGCAGACAAGTACTTTCAC-3'	5'- GGCCGTCACTGATCACTTCAG-3'
tf	5'-TGCCGTCTCCAGGTTCTTTT-3'	5'-GGGCACAGTTGGCACACA-3'
